# Phosphorylation of birch BpNAC90 improves the activation of gene expression to confer drought tolerance

**DOI:** 10.1093/hr/uhae061

**Published:** 2024-02-28

**Authors:** Zhibo Wang, Zihang He, Caiqiu Gao, Chao Wang, Xingshun Song, Yucheng Wang

**Affiliations:** College of Life Science, Northeast Forestry University, Harbin 150040, China; State Key Laboratory of Tree Genetics and Breeding, Northeast Forestry University, Harbin 150040, China; State Key Laboratory of Tree Genetics and Breeding, Northeast Forestry University, Harbin 150040, China; State Key Laboratory of Tree Genetics and Breeding, Northeast Forestry University, Harbin 150040, China; State Key Laboratory of Tree Genetics and Breeding, Northeast Forestry University, Harbin 150040, China; College of Life Science, Northeast Forestry University, Harbin 150040, China; State Key Laboratory of Tree Genetics and Breeding, Northeast Forestry University, Harbin 150040, China; State Key Laboratory of Tree Genetics and Breeding, Northeast Forestry University, Harbin 150040, China

## Abstract

The NAC transcription factors (TFs) play important roles in mediating abiotic stress tolerance; however, the mechanism is still not fully known. Here, an *NAC* gene (*BpNAC90*) from a gene regulatory network of *Betula platyphylla* (birch) that responded to drought was characterized. Overexpression and knockout of *BpNAC90* displayed increased and reduced drought tolerance, respectively, relative to wild-type (WT) birch. BpNAC90 binds to different DNA motifs to regulate target genes in conferring drought tolerance, such as Eomes2, ABRE and Tgif2. BpNAC90 is phosphorylated by drought stress at Ser 205 by birch SNF1-related protein kinase 2 (BpSRK2A). Mutated *BpNAC90* (termed *S*^*205*^*A*) with abolished phosphorylation, was transformed into birch for overexpression. The transgenic *S*^*205*^*A* plants displayed significantly reduced drought tolerance compared with plants overexpressing *BpNAC90*, but still showed increased drought tolerance relative to WT birch. At the same time, S^205^A showed a decreased capability to bind to motifs and reduced activation of target gene expression, which contributed to the reduced drought tolerance. Additionally, BpSRK2A and BpNAC90 can be induced by drought stress and form a complex to phosphorylate BpNAC90. The results together indicated that phosphorylation of BpNAC90 is necessary in conferring drought tolerance in birch.

## Introduction

The family of NAM, ATAF, and CUC (NAC) proteins constitutes one of the largest transcription factor families [1]. NAC TF family includes a conserved NAC domain-petunia NAM and *Arabidopsis thaliana* ATAF1, ATAF2, and CUC2 (cup-shaped cotyledon), located at N-terminal region [2]. These conserved NAC domains bind to both DNA and other proteins [3]. To date, many NAC proteins had been discovered in the genomic sequences of different plant species, including 75 in rice (*Oryza sativa*) [4], 189 in *Eucalyptus grandis* [5], 105 in Arabidopsis [4], and 170 in *Populus deltoides × Populus euramericana* [6].

NAC TF plays a role in different biological processes, including plant development and growth [7–10]. For instance, tomato non-ripening like1 (SlNOR), an NAC TF, could activate encoding1-aminocyclopropane-1-carboxylic acid synthase2 (*SlACS2*) by binding to its promoter to regulate fruit ripening [11]. Overexpression of *OsSNAC1* in wheat increased the sensitivity to abscisic acid (ABA), leading to high levels of water and chlorophyll in the leaves, and inhibition of root and shoot growth [12].

At the same time, NAC TFs have made important contributions to improving the viability of plants under adverse conditions. Overexpression of rice *SNAC2* (*OsSNAC2*) in transgenic rice significantly improved germination and growth relative to those of wild-type (WT) plants when exposed to high salinity [13]. Overexpression of *OsSNAC1* in *Gossypium hirsutum* improved drought and salt resistance by promoting more vigorous growth of the roots and reducing the transpiration rate of transgenic plants [14]. Overexpression of *OsNAC066* in transgenic rice enhanced their tolerance to oxidative and drought stress, decreased water loss rate and reactive oxygen species (ROS) accumulation. In addition, the expression of stress-associated genes was induced under drought stress conditions [15]. Overexpression of *SNAC1* conferred significantly drought and salinity tolerance with elevated chlorophyll and water level [12]. Similarly, transgenic Arabidopsis plants overexpressing (OE) *GmNAC019* displayed higher survival rates under soil-drying conditions, accompanied by a reduced water loss rate and cellular hydrogen peroxide (H_2_O_2_) content, and increased antioxidant defense [16]. Overexpression of *MuNAC4*, an *NAC* gene from *Macrotyloma uniflorum*, conferred drought tolerance by enhancing lateral roots, decreasing the damage to membrane structures, and increasing osmotic adjustment and antioxidative enzyme activities [17]. Like other TFs, NAC TFs regulate a series of genes associated with abiotic stress to facilitate stress tolerance. For instance, GmNAC11 could regulate Arabidopsis the *DREB1A* (encoding drought response element binding 1A), *ERD11* (encoding early dehydration response 11), *COR15A* (encoding cold regulation 15A), *ERF5* (encoding ethylene response factor 5), *RAB18* (encoding ras-related protein Rab18), and *KAT2* (encoding potassium channel 2) genes to improve salt tolerance [18]. Overexpression of *CarNAC4* in Arabidopsis plants increased expression of stress-associated genes, including *KIN1* (encoding kinesin-like protein 1), *RD29A* (encoding RESPONSIVE TO DESICCATION 29A), *ERD10*, *COR15A*, *COR47*, and *DREB2A* to mediate drought and drought stress response [19].

Previous studies showed that plants respond to an adverse environment in two approaches (i.e., protein modification and expression regulation) [20]. Post-translational modifications (PTMs), including glycosylation, methylation, ubiquitination, acetylation, phosphorylation and other modifications, play critical roles in the modulation of protein function [21]. Among PTMs, dephosphorylation and phosphorylation mediated by protein kinase are important PTMs [22]. For instance, SnRKs play roles in protein phosphorylation and play roles in stress tolerance [23]. SOS3 (salt overly sensitive 3; also known as AtCBL4) can recognize the calcium signal on the cell membrane induced by drought stress, and then SOS2 (also known as AtCIPK24) joins with SOS3 for phosphorylating SOS1 (Na^+^/H^+^ antiporter) to remove excess sodium ion from cells [24, 25]. SnRK2.10 can phosphorylate different proteins, such as dehydrins, early response to dehydration 14 (ERD14) and ERD10, to respond to osmotic stress [26]. Apple CBL-interacting protein kinase 22 (MdCIPK22) and MdCIPK13 are found to phosphorylate MdSUT2.2, the sucrose transporter in response to drought stress [27, 28]. Phosphorylation modification modulates the activities of TF by regulating their DNA-binding affinity, transactivation, stability, and distribution [29]. The secondary cell-wall regulator NST1 can interact with SnRK2.2.3 to phosphorylate NST1 at Ser316, leading to inducing a serial of genes related with the biosynthesis of cellulose and lignin [30]. ZmNAC84 can be phosphorylated at Ser-113, and phosphorylation of ZmNAC84 could improve tolerance to drought of *Zea mays* by increasing the binding affinity of ZmNAC84 to the promoter of *ZmSOD2* for up-regulating the *ZmSOD2* expression [31]. Although NAC TFs had been studied intensively, there are still some issues that need to be clarified, such as can NAC also be regulated in response to abiotic stress by PTMs? If so, what are the mechanisms? Furthermore, what is the mechanism of phosphorylation of NAC TFs? In addition, NAC TFs bind to various *cis*-acting elements to perform their diverse functions; therefore, which *cis*-acting elements do they mainly bind to mediate abiotic tolerance?

Our previous study built a gene regulatory network (GRN) for *Betula platyphylla* (birch) under drought conditions [32]. *BpNAC90* was at the top level of the GRN, suggesting that it might play an important role in the drought response. In the present investigation, we used RNA sequencing (RNA-seq) and Chromatin immunoprecipitation sequencing (ChIP-seq) to determine the genes directly or indirectly regulated by BpNAC90. Furthermore, the DNA motifs recognized by BpNAC90 were revealed. We found that phosphorylation of BpNAC90 responding to drought stress enhances its capability to regulating the downstream genes, thereby enhancing drought tolerance. Moreover, the mechanism of phosphorylation of BpNAC90 was revealed. These findings provide useful data for revealing the function of BpNAC90.

## Results

### Generation of birch plants with *BpNAC90* OE or knockout

**Figure 1:**
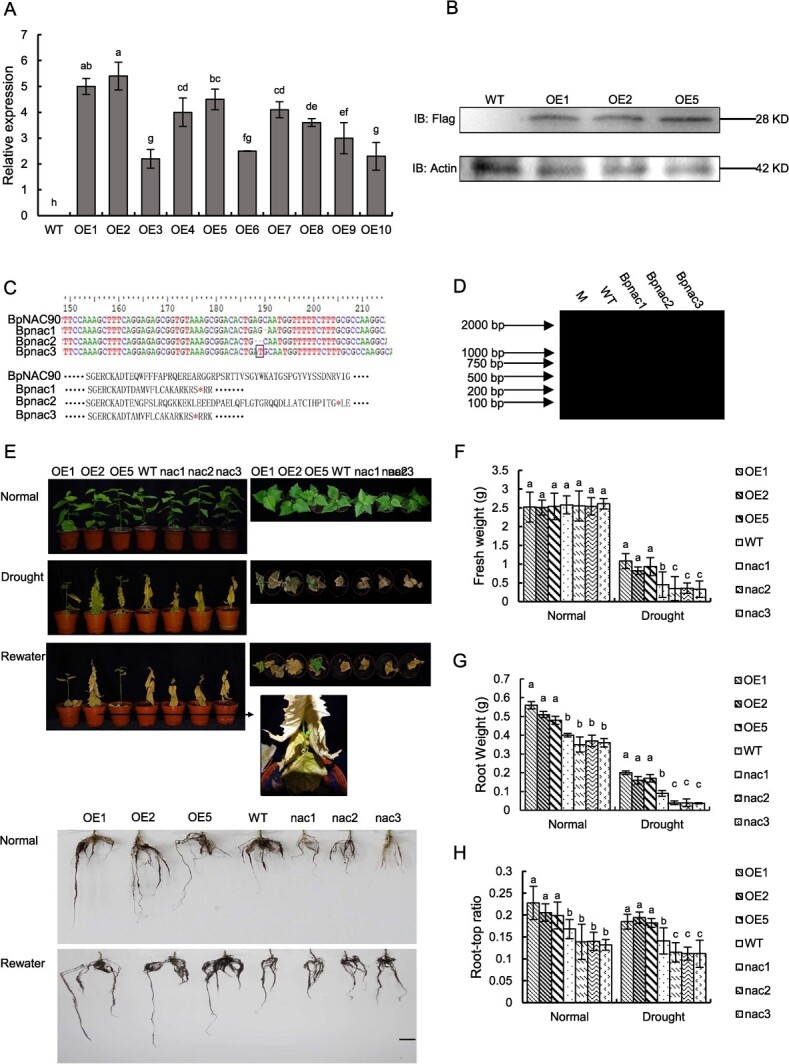
Generation of *BpNAC90* OE and knockout birch plants, and analysis of their tolerance to drought stress. **A** Determination of the expression of *BpNAC90* in transformed lines using qRT-PCR. The expression level of *BpNAC90* in WT plants was set as 1 to calculate the relative expression of *BpNAC90* in other lines, and the relative expression values were log2 transformed. >0 and <0, respectively, indicate the expression of *BpNAC90* in lines were higher or lower than that in WT plants. **B** Determination of the expression of the Flag-*BpNAC90* fusion protein using western blotting with an anti-Flag antibody. **C** Analysis of the mutation of the BpNAC90 sequence induced by CRIPSR-editing. **D** T7E1 digestion to analyse the homozygous and heterozygous mutation of the CRISPR edited lines. **E** The growth phenotype of the BpNAC*90* OE and knockout birch lines. Scale bars, 10 cm. **F**–**H** Comparison of fresh weight (**F**), root weight (**G**), and root-top ratio (**H**) analysis among the studied birch lines under normal or drought stress conditions. The experiment was performed three times with similar results. Error bar indicates standard deviation (SD) from the three experiments. a–h indicate multiple comparison difference (LSD - *t* test, 0.05). OE1, 2 and 5: birch lines overexpressing *BpNAC90*; WT: wild-type birch; *nac1–3*: the plants with mutated *BpNAC90* induced by CRISPR/Cas9 birch lines.

In previous research, a birch GRN responding to drought was constructed [32]. BpNAC90 was found in the top level of GRN, and kits encoded protein regulated several TFs, and these TFs then regulate a series of structure genes in response to drought stress. These results suggested that BpNAC90 might play an important role in drought tolerance. To investigate the functions of BpNAC90, the birch plants with *BpNAC90* OE and knockout (CRISPR edited plants, termed *nac* lines) were respectively generated. The transgene expression was determined by qRT-PCR, and the results showed that the expression levels of *BpNAC90* were significantly elevated in OE plant lines relative to that in WT birch (Fig. 1A). The lines OE1, OE2, and OE5 were selected for western blotting assay. Western blotting indicated that BpNAC90 protein was successfully expressed in these OE lines (Fig. 1B). Sanger sequencing was performed to study the CRISPR editing birch plants, and there were three lines mutated with an open reading frame shift (Fig. 1C), but they may be heterozygous for the reason that the WT sequence was also detected. In addition, T7EI analysis showed that these three mutations were all heterozygous; for the reason that the band of each line can be cleaved, which suggested that the cleaved bands were constituted with WT sequence and mutation sequences to form a loop digested by T7EI (Fig. 1D). These mutated lines were termed *nac1*, *nac2*, and *nac3*, and were selected for subsequent study.

### *BpNAC90* confers drought stress tolerance

To study the drought tolerance function of *BpNAC90*, the growth phenotype of OE lines (1, 2, and 5), *nac* lines (1, 2, and 3), and WT plants were compared (Fig. 1E). All the lines studied displayed similar growth phenotypes under normal conditions. However, under drought stress conditions, all *nac* lines displayed decreased plant heights and fresh weights, reduced root weights and decreased root-top ratios relative to the control plants (WT); however, all OE lines displayed improved plant heights and fresh weights, enhanced root weights and root-top ratios relative to the WT birch (Fig. 1F–H). In addition, after rehydration for 12 days, all the OE plants treated (each line contains three plantlets, and nine plantlets in total) had recovered to a live state, with a survival rate of 100%; however, the *nac* lines and WT plants all died (Fig. 1E). These results together indicated that *BpNAC90* confers drought tolerance to birch.

### Overexpression of *BpNAC90* reduced cell death and increased the proline content and ROS scavenging capability

**Figure 2:**
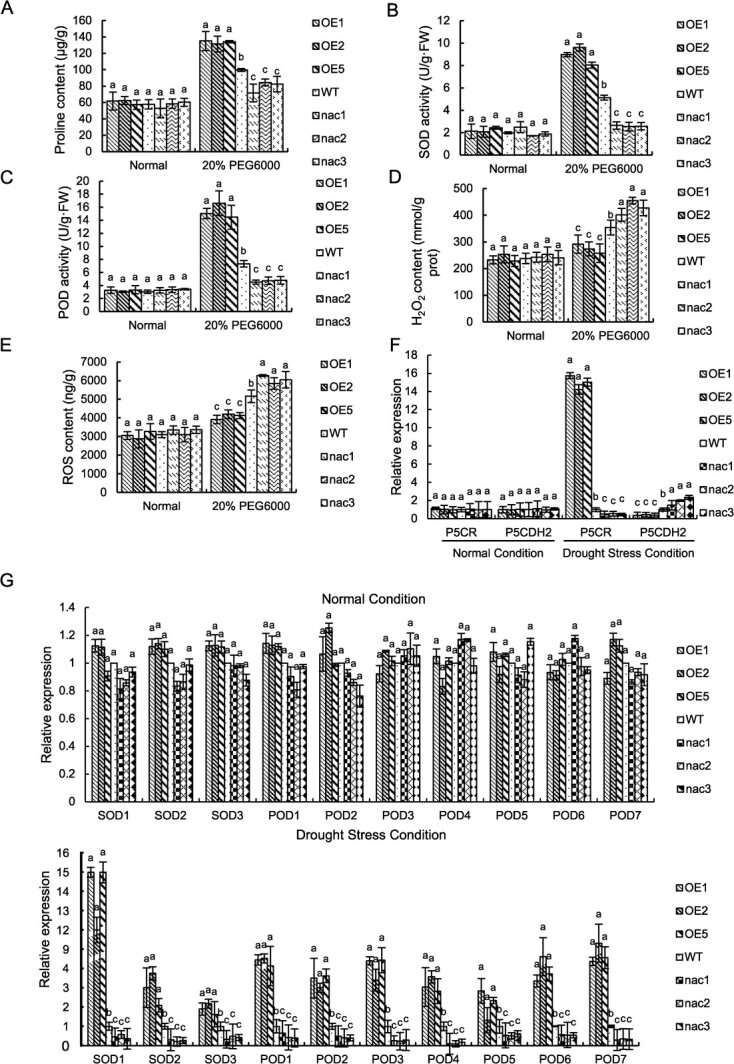
Analysis of proline content and ROS scavenging capability. **A** Proline content analysis. **B**, **C** Analysis of the activities of SOD (**B**) and POD (**C**). **D**, **E** Analysis of the contents of H_2_O_2_ (**D**) and ROS (**E**). **F**, **G** Comparison of the expression of genes related to proline biosynthesis and metabolism (**F**), and *SODs* and *PODs* (**G**) among the WT and the *BpNAC90* OE and knockout birch lines. The experiment was performed three times with similar results. Error bar indicates standard deviation (SD) from the three experiments. a, b, and c indicate multiple comparison difference (LSD - *t* test, 0.05).

Under normal conditions, no significant difference in both malondialdehyde (MDA) content and electrolyte leakage rate was found among the studied plant lines (Fig. S1A and B, see online supplementary material). Under drought conditions, both the electrolyte leakage and MDA content were significantly reduced in all OE plants, but significantly increased in all *nac* plants, relative to the control plants (WT) (Fig. S1A and B, see online supplementary material). Under normal conditions, the proline contents in all the lines studied were similar. Under drought treatment conditions, proline content was elevated significantly in all the OE plants, but in the *nac* lines, the proline contents were significantly decreased relative to those in the WT plants (Fig. 2A). The expression of genes related to proline biosynthesis (*P5CR*, encoding δ-pyrroline-5-carboxylate reductase) and metabolism (*P5CDH2*, encoding Delta-1-pyrroline-5-carboxylate dehydrogenase) were examined using qRT-PCR. Under drought treatment, *BpNAC90* significantly induced the expression of *P5CR* and decreased *P5CDH2* expression (Fig. 2F). The results together indicated that *BpNAC90* could induce the genes associated with proline biosynthesis and inhibit the genes involved in metabolism to accumulate proline, thus enhancing drought tolerance.

Under normal conditions, both superoxide dismutase (SOD) and peroxidase (POD) activities were similar among all the plants studied. Under drought treatment conditions, the activities of SOD and POD were both improved significantly in all OE lines but were both significantly reduced in all *nac* lines relative to WT plants (Fig. 2B and C). Correspondingly, both ROS and H_2_O_2_ levels in OE lines were significantly lower than those in the WT plants, but in the *nac* lines, they were significantly higher than those in the WT plants (Fig. 2D and E). Furthermore, the expression of *SODs* and *PODs* (including *POD1*-*POD7* and *SOD1*-*SOD3*) were not differentially regulated by *BpNAC90* under normal conditions, but were significantly induced by *BpNAC90* (Fig. 2G), which contributed to the increased POD and SOD activities. The results together indicated that overexpression of *BpNAC90* could increase activities of POD and SOD through increasing the transcription of *PODs* and *SODs*, thereby enhancing the capability of ROS scavenging.

### BpNAC90 could bind to Eomes2, ABRE, and Tgif2 motifs of gene promoters to mediate drought tolerance

**Figure 3:**
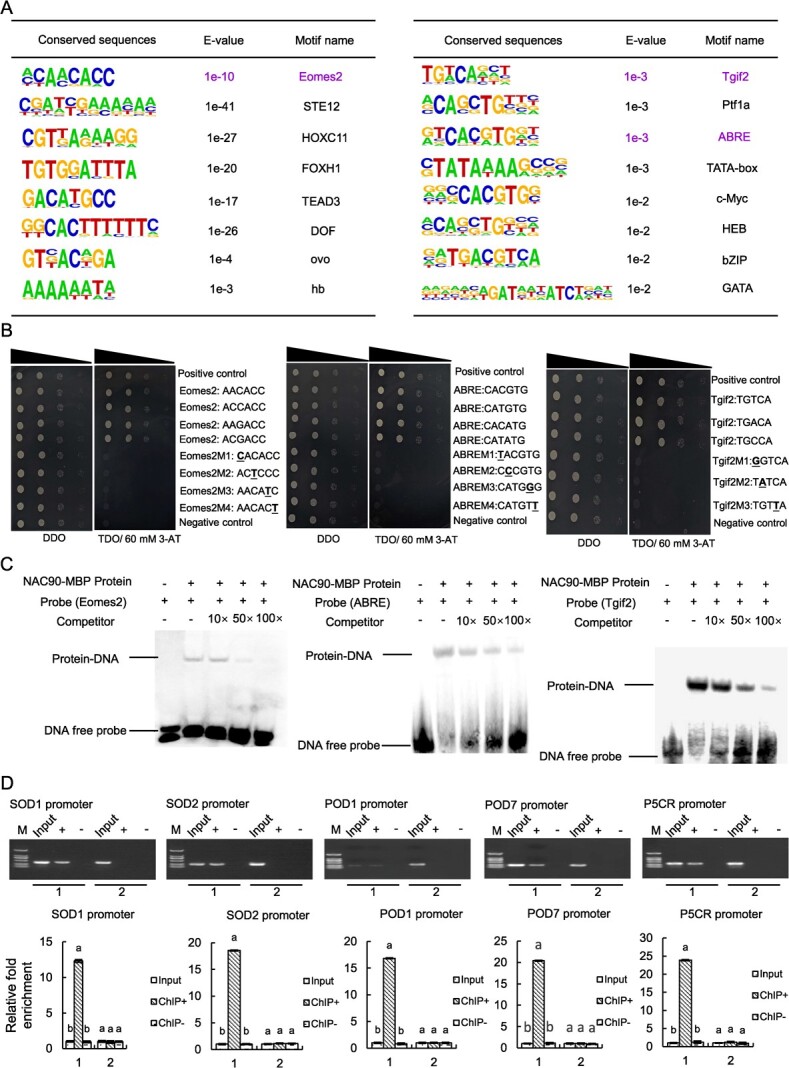
Determination of the DNA motifs potentially bound by BpNAC90. **A** The DNA motifs potentially bound by BpNAC90 and predicted by ChIP-Seq. Pink color motifs: the DNA motifs bound by BpNAC90. **B** Analysis of the binding of BpNAC90 to the DNA motifs using Y1H. Yeast transformants were spotted using serial dilutions (1, 10^−1^, 10^−2^, 10^−3^). **C** Determination of the binding of BpNAC90 to the motifs using EMSA analysis. **D** The binding of BpNAC90 to the promoters of *POD*, *SOD*, and *P5CR* genes containing DNA motifs by ChIP-PCR and ChIP-qPCR. 1: the promoter region of genes containing DNA motifs; 2: the promoter region of genes not containing DNA motifs. Fold enrichment: the amount of aim DNA region relative to that of internal control DNA region, and fold enrichment in Input is set as 1 to calculate the relative fold enrichment in ChIP+ and ChIP-. Three biological replicates were performed. Error bar indicates standard deviation (SD) from the three experiments. a and b indicate multiple comparison difference (LSD - *t* test, 0.05).

The ChIP-Seq was performed using Flag*-BpNAC90* overexpressing birch, and the input DNA was employed to remove the non-specific peaks. In total, 27 522 specific peaks that were potentially bound by BpNAC90 were identified. Among them, there were 23.11% of the peaks distributed in the promoter area (Fig. S2A, see online supplementary material). The 9305 genes with the peaks distributed in their promoters were determined (see Table S1, see online supplementary material). Among these genes, 5955 genes had been annotated by GO analysis. GO classification indicated that purine ribonucleoside triphosphate binding, protein modification by small protein removal, cation transmembrane transporter activity, drug binding, ATP binding, and so on were highly enriched (Fig. S2B, see online supplementary material). Motif discovery analysis was conducted to detect the conserved DNA sequences potentially bound by BpNAC90, and 16 conserved motifs were identified (Fig. 3A). To investigate whether BpNAC90 binds to these motifs, three motifs were randomly selected, including Eomes2 (‘AACACC’), ABRE (‘CACGTG’), and Tgif2 (‘TGTCA’) for yeast one hybrid (Y1H) verification (Fig. 3B). Eomes2, ABRE, and Tgif2 sequences could be bound by BpNAC90, but their mutants were not bound by BpNAC90 (Fig. 3B). Furthermore, electrophoretic mobility shift assay (EMSA) showed that all three motifs could retard DNA-protein band mobility, which were all gradually decreased with the increase in the unlabeled competitor probe concentration (Fig. 3C), further confirming that BpNAC90 binds to Eomes2, ABRE, and Tgif2 motifs.

To determine whether BpNAC90 can bind to the identified DNA motifs to regulate gene expression, ChIP-PCR and ChIP-qPCR were performed. The genes for *POD*, *SOD*, and *P5CR* whose promoters containing Eomes2, ABRE, and/or Tgif2 motifs were induced by BpNAC90 (Fig. 3D) were analysed. The results indicated that BpNAC90 can bind to these DNA motifs, suggesting that BpNAC90 binds to these DNA motifs to regulate gene expression.

RNA-seq was performed on WT and OE1 lines to identify the target genes of BpNAC90. In total, 352 differentially expressed genes (DEGs) had been determined, including 192 genes that were induced and 160 that were inhibited by BpNAC90 (Table S2, see online supplementary material). Conjoint analysis of RNA-seq and ChIP-Seq was performed, and 117 genes were identified, including 66 downregulated genes and 51 upregulated genes (Table S3, see online supplementary material). To further verify the reliability of conjoint analysis results, ChIP-PCR and ChIP-qPCR were performed. Five genes whose promoters contain Eomes2, ABRE, and Tgif2 motifs were selected for study, and these genes included *Bp5G13007* (Galactinol synthase 1), *Bp1G24271* (Pollen-specific leucine-rich repeat extensin-like protein), *Bp12G11354* (Protein MIZU-KUSSEI 1-like), *Bp9G16353* (FAD-binding domain) and *Bp8G16690* (ATP binding protein) (Table S3, see online supplementary material). These results indicated that the genes with Eomes2, ABRE, and Tgif2 sequences in their promoters could all be significantly enriched by BpNAC90 (Fig. S3A and B, see online supplementary material), confirming that the conjoint analysis results are reliable. In addition, the bindings of BpNAC90 to Eomes2, ABRE, and Tgif2 motifs were also confirmed by dual luciferase (LUC) reporter assay (Fig. S3C, see online supplementary material), suggesting that BpNAC90 regulates gene expression through binding to these three motifs.

The above five genes bound by BpNAC90 were separately constructed into pROKII and pFGC5941 vectors for overexpression and RNAi-silenced expression (IE), and then transiently transformed into birch plants for drought tolerant measurement. The physiological indexes of these five genes were all similar among control, OE, and IE plants without stress treatment. Under drought treatment conditions, the OE plants for these five genes had the lowest electrolyte leakages, MDA contents, and ROS accumulation, followed with the controls; while the IE plants had the highest electrolyte leakages, MDA contents, and ROS accumulation, suggesting that these genes could confer drought tolerance (Fig. S4, see online supplementary material).

### Phosphorylation of BpNAC90 was induced by drought stress

**Figure 4:**
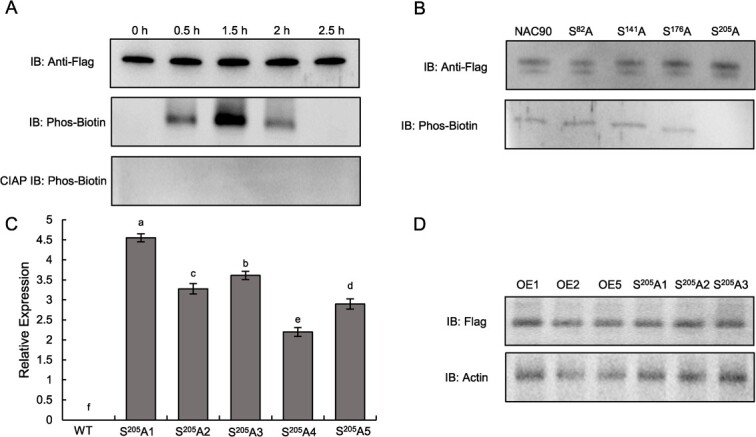
BpNAC90 is phosphorylated by drought. **A** Analysis of the phosphorylation modification of BpNAC90 in response to drought treatment. **B** Determination of the phosphorylated site of BpNAC90 protein. The potentially phosphorylated serine was mutated to alanine to detect whether it can be phosphorylated. S^N^A: S indicates serine; N indicates the position of serine; A indicates serine mutated to alanine. Phos-Biotin: biotin-labeled Phos-tag, which was used for western blotting analysis. CIAP IB: the BpNAC90 protein was treated with calf intestine alkaline phosphatase to remove the phosphorylation modification, which served as negative control. **C** Determination of the expression of *S^205^A* in birch transformed lines. S^205^A: The serine at 205 position was mutated to alanine to abolish the phosphorylation modification of BpNAC90. The expression level of *S^205^A* in WT plants was set as 1 to calculate the relative expression of other *S^205^A* lines, and the relative expression values were log2 transformed. **D** Western blotting analysis of the expression of S^205^A protein in S^205^A1–3 lines and the expression of transformed *BpNAC90* in OE1, 2 and 5 lines. The experiment was performed three times with similar results. Error bar indicates standard deviation (SD) from the three experiments. a–f indicate multiple comparison difference (LSD - *t* test, 0.05).

For identifying whether the phosphorylation of BpNAC90 is responding to drought stress, the BpNAC90 protein was immunoprecipitated from the OE line with anti-Flag antibody under normal and drought treatment conditions for different periods. The immunoprecipitated BpNAC090 was separated by SDS-PAGE, and its phosphorylation was identified by Phos-tag Biotin. The phosphorylation level of BpNAC90 is elevated from 0.5 to 1.5 h, peaking at 1.5 h, and then was reduced gradually after 1.5 h of drought stress (Fig. 4A). The above results suggested that BpNAC90 phosphorylation was induced by drought stress.

To further study the phosphorylation site of BpNAC90, 4 potential phosphorylated sites (Ser^82^, Ser^141^, Ser^176^, and Ser^205^) were studied. The serine residues of Ser^82^, Ser^141^, Ser^176^, and Ser^205^ were respectively substituted with alanine (termed as S^82^A, S^141^A, S^176^A, and S^205^A correspondingly). These mutated *BpNAC90s* were separately fused with Flag tag sequence and were separately transiently transformed into the WT birch for overexpression. The transformed birch plants were grown on a medium containing 20% polyethylene glycol (PEG) 6000 for 1.5 h, and the mutated proteins of BpNAC90 were respectively immunoprecipitated with the anti-Flag antibody. The phosphorylation of S^82^A, S^141^A, S^176^A, and S^205^A proteins were detected using western blotting with biotinylated Phos-tag. Only the S^205^A mutation could abolish phosphorylation among these mutation proteins, indicating that in BpNAC90, Ser^205^ is the only phosphorylation site (Fig. 4B).

### Function of phosphorylation of BpNAC90 in response to drought stress

**Figure 5:**
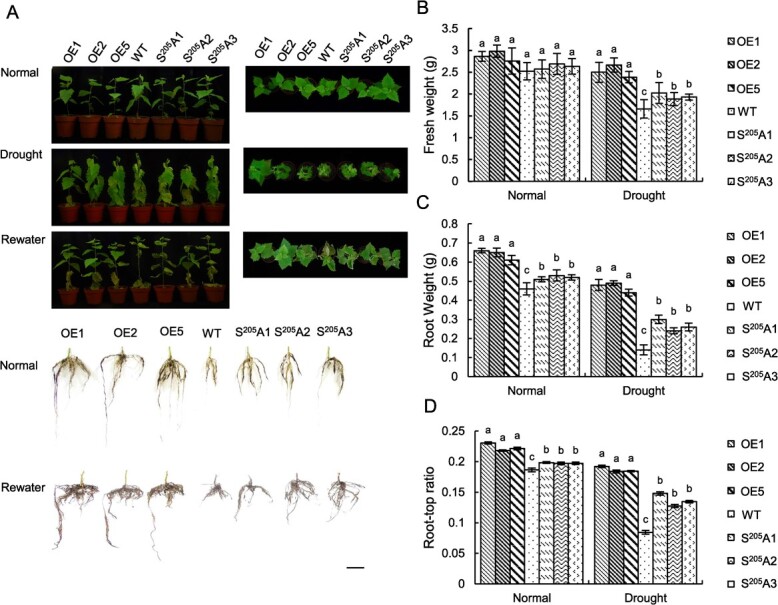
The mutated BpNAC90 abolishing phosphorylation modification (S^205^A) reduced drought tolerance conferring. **A** The growth phenotype of the *BpNAC90* OE and knockout birch lines. Scale bars, 10 cm. **B**–**D** Comparison of the fresh weight (**B**), root weight (**C**), and root-top ratio (**D**) among the studied birch lines under normal or drought stress conditions. OE1, 2, and 5: The birch lines overexpressing *BpNAC90*; WT: wild-type birch; *S^205^A1*–*3*: The birch lines (1–3) transformed with *S^205^A* for overexpression. The experiment was performed three times with similar results. Error bar indicates standard deviation (SD) from the three experiments. a–c indicate multiple comparison difference (LSD - *t* test, 0.05).

The mutation of *BpNAC90, S^205^A*, was stably transformed into WT birch for overexpression, and was used for investigating the function of phosphorylation. Three transgenic lines (termed as S^205^A1–3) were selected for analysis (Fig. 4C). We first compared the expression of S^205^A and BpNAC90 in the corresponding transgenic lines. Western blotting showed that the expressions of S^205^A protein in S^205^A1–3 lines were similar to those of transgenic BpNAC90 protein in OE lines (Fig. 4D). All the plants shared similar fresh weights, growth phenotypes, and root-top ratios under normal conditions. Under drought treatment condition, *S^205^A* lines displayed significant higher fresh weights, root weights, and root-top ratios than WT plants, but showed significant lower fresh weights, root weights, and root-top ratios than the OE lines (Fig. 5A–D). After rewatering for 10 days, the OE lines had recovered to almost fully alive conditions, followed by *S^205^A* lines, and then the WT plants (Fig. 5A).

Physiological analysis showed that all OE lines displayed the lowest electrolyte leakages, MDA content, H_2_O_2_ content, and ROS content under drought conditions, the *S^205^A* lines came second, and followed by the WT plants (Fig. S5A–D, see online supplementary material); however, all the studied plants displayed similar electrolyte leakages, MDA content, H_2_O_2_ and ROS content under normal conditions (Fig. S5A–D, see online supplementary material). Moreover, proline contents and the activities of SOD and POD in all the plants studied were similar under normal conditions. However, *S^205^A* lines had lower proline contents and the activities of POD and SOD than OE lines had but displayed higher POD and SOD activities and proline contents than WT plants under drought treatment conditions (Fig. S5E–G, see online supplementary material). The results together suggested that abolishing phosphorylation of BpNAC90 reduced drought stress tolerance, but still retained some drought tolerance.

### Non-phosphorylated BpNAC90 displayed reduced activation on gene expression

**Figure 6:**
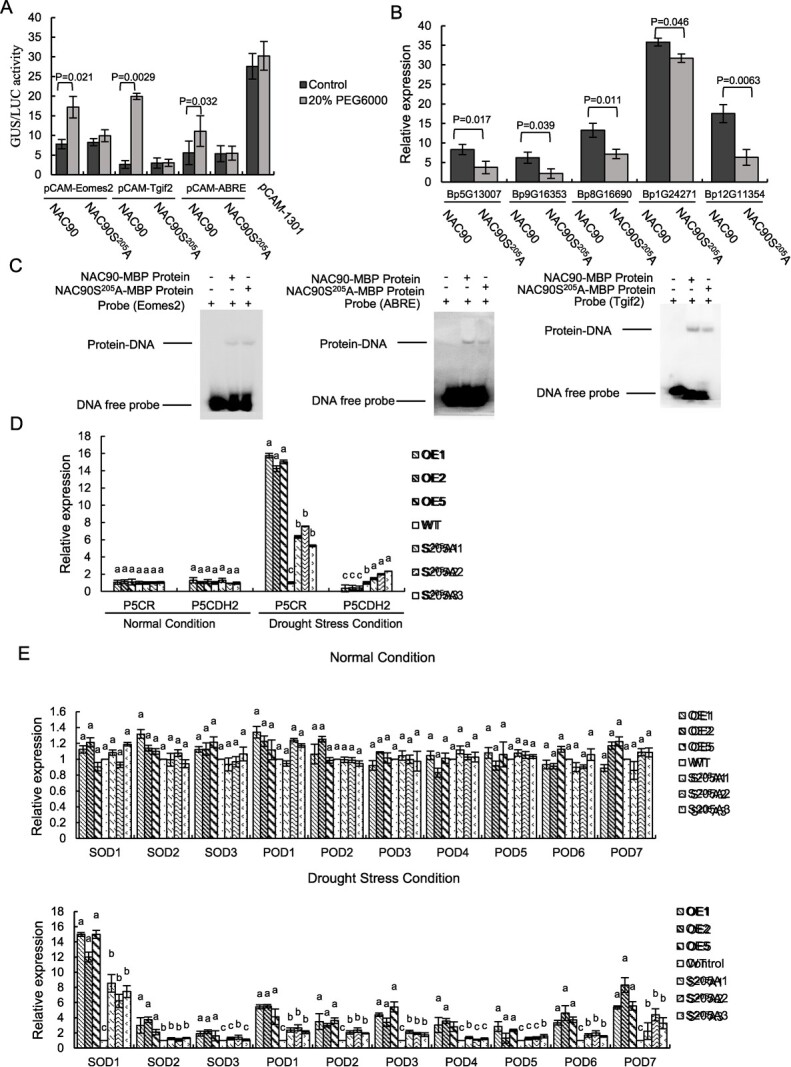
Comparison of *BpNAC90* and *S^205^A* on activation of gene expression. **A** Comparison of the activation of gene expression between *BpNAC90* and *S^205^A* when binding to different DNA motifs. Three tandem copies of the motifs were fused with the 46-bp minimal 35S promoter to drive a *GUS* gene in a reformed pCAMBIA1301 vector (from which the region of 35S:Hygromycin was removed and a 46-bp minimal promoter was inserted) as reporters. The CDS of *BpNAC90* or *S^205^A* was respectively cloned into pROKII under the control of 35S promoter as effectors. The construct of 35S:*Luc* was also co-transformed to normalize the transformation efficiency. **B** Comparison of the expression levels of genes regulated by *BpNAC90* and *S^205^A* under drought conditions. According to conjoint analysis of ChIP-seq and RNA-seq, five genes whose promoters are bound by BpNAC90 to regulate them were selected for study. **C** The binding of BpNAC90 and S^205^A to Eomes2, ABRE, and Tgif2 by EMSA assay. Both of BpNAC90 and S^205^A protein were prokaryotically expressed and purified, and used for EMSA. **D** Comparison of the expression levels of genes related to proline biosynthesis and metabolism regulated by *BpNAC90* and *S^205^A*. **E** Comparison of the expression levels of *PODs* and *SODs* regulated by *BpNAC90* and S*^205^A*. The experiment was performed three times with similar results. Error bar indicates standard deviation (SD) from the three experiments. *P* values were derived by Student’s *t* test and a, b, and c indicate multiple comparison difference (LSD - *t* test, 0.05).

*BpNAC90* and its mutation *S^205^A* were respectively cloned into pROKII vector driven by 35S promoter (effectors). Eomes2, ABRE, or Tgif2 motif with three tandem copies was fused with a 46-bp minimal 35S CaMV promoter to drive a *GUS* gene, and served as reporters. The effector, reporter, and 35S:*Luc* gene driven by 35S promoter was used to normalize transformation efficiency constructs and were together transiently transformed into birch plants. These transgenic plants were treated with drought induced by PEG6000 and the plants without drought stress were served as the control. When binding to the Eomes2, ABRE, or Tgif2 motif, BpNAC90 can activate gene expression more significantly than *S^205^A* did (Fig. 6A). In addition, the gene expression activated by *BpNAC90* (when binding to Eomes2, ABRE, or Tgif2) could be induced by drought, but that mediated by *S^205^A* cannot be induced by drought (Fig. 6B), indicating that phosphorylation of Ser^205^ plays an important role in gene expression activation, and is also necessary to respond to drought stress. We further performed EMSA to study the binding of BpNAC90 and S^205^A protein to these three DNA motifs; both were prokaryotically expressed and purified for EMSA. The results showed that both BpNAC90 and S^205^A protein can bind to Eomes2, ABRE, and Tgif2 (Fig. 6C), suggesting that BpNAC90 without phosphorylation could still bind to these motifs. Correspondingly, the expression of the five target genes (*Bp5G13007*, *Bp1G24271*, *Bp12G11354*, *Bp9G16353*, and *Bp8G16690*) identified by conjoint analysis of RNA-seq and ChIP-Seq; *PODs*, *SODs*, *P5CR*, and *P5CDH2* were compared between OE and *S^205^A* lines using qRT-PCR. The expression of all the genes studied were not differentially regulated among the studied plants under normal conditions; however, these genes were significantly decreased in the *S^205^A* lines compared with in the OE lines; however, their expression levels in the *S^205^A* lines were all significantly higher than those in the WT plants except *P5CDH2* (relative to proline metabolism) (Fig. 6D and E), suggesting that phosphorylation of BpNAC90 is necessary to activate gene expression under drought stress conditions.

### SNF1-related protein kinase 2 (BpSRK2A) interacts with BpNAC90 to phosphorylate it

**Figure 7:**
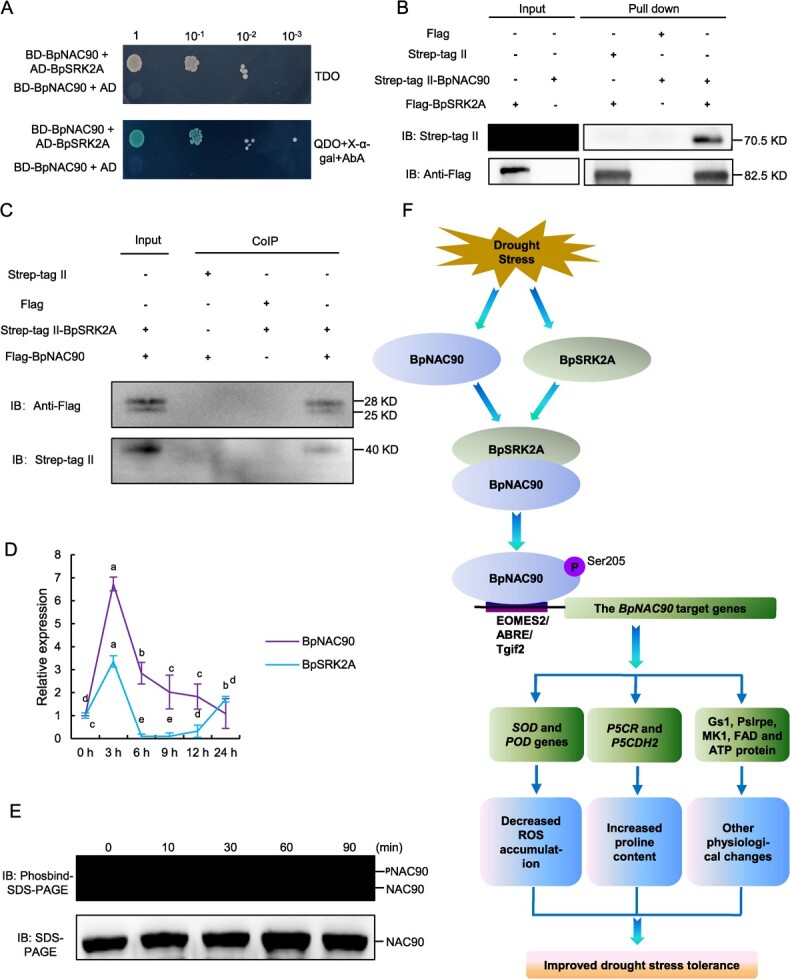
BpSRK2A interacts with and phosphorylate BpNAC90 and the working model of how BpNAC90 modulates drought stress. **A** Verification of the interaction between BpNAC90 and BpSRK2A using the Y2H system. The interaction of BpSRK2A with BpNAC90 was identified by screening cDNA library of Y2H. Yeast transformants were spotted using serial dilutions (1, 10^−1^, 10^−2^, 10^−3^). **B** Verification of the interaction between BpNAC90 and BpSRK2A using a protein pull-down assay. **C** Verification of the interaction between BpNAC90 and BpSRK2A using CoIP. The fusion genes Flag-*BpNAC90* and Strep tagII-*BpSRK2A* were transiently transformed into birch together and immunoprecipitated using anti-Flag antibodies. The IP product was analysed using western blotting using anti-Flag antibodies and anti-Strep tagII antibodies, respectively. **D** The expression of *BpNAC90* and *BpSRK2A* in response to drought stress. The experiment was performed three times with similar results. Error bar indicates standard deviation (SD) from the three experiments. a–e indicate multiple comparison difference (LSD - *t* test, 0.05). **E** Verification of the phosphorylation of BpNAC90 medicated by BpSRK2A. Both BpNAC90 and BpSRK2A were prokaryoticly expressed and purified. The purified BpNAC90 and BpSRK2A were incubated together for different times and then the phosphorylation of BpNAC90 was detected using Phosbind. ^p^NAC90: phosphorylated BpNAC90 protein; NAC90: BpNAC90 protein. **F** The working model of how BpNAC90 modulates drought stress. *BpNAC90* and *BpSRK2A* expressions are induced by drought stress, and the induced BpNAC90 and BpSRK2A form a complex to phosphorylate BpNAC90 at serine 205. The phosphorylated BpNAC90 then binds to the DNA motifs such as EOMES2, ABRE, and Tgif2 to regulate gene expression, which will induce the expression of genes encoding proteins involved in ROS scavenging, proline biosynthesis, and metabolism, and other genes related to drought stress (identified by RNA-seq). The regulation of these genes leads to decreased ROS levels and an increased proline content, which improve drought tolerance.

To identify the protein that interacts with BpNAC90, BpNAC90 was cloned into pGBKT7 and used as bait protein to screen a cDNA library of birch via a yeast two hybrid (Y2H) screen. After Y2H screening and Sanger sequencing, BpSRK2A was identified. To further verify the interaction of BpSRK2A and BpNAC90, Y2H was further performed (Fig. 7A). The pull-down experiment showed that BpNAC90 fused with a Strep tagII tag could be pulled down by BpSRK2A fused with a Flag tag (Fig. 7B), confirming that BpSRK2A interacts with BpNAC90. In addition, in the co-immunoprecipitation (CoIP) analysis, when BpNAC90-Flag was immunoprecipitated using anti-Flag antibodies, the BpSRK2A-Strep tagII in the immunoprecipitation (IP) product could be detected by western blotting using anti-Strep tagII antibodies (Fig. 7C), suggesting that BpSRK2A and BpNAC90 can interact with each other in birch plants.

The expressions of *BpNAC90* and *BpSRK2A* responding to drought stress were studied, and showed similar expression profiles, both reaching a peak after drought stress for 3 h, after which their expression levels were highly reduced (Fig. 7D). We further determined whether BpNAC90 could be phosphorylated by BpSRK2A. Both BpNAC90 and BpSRK2A were prokaryoticly expressed and then purified. The purified BpNAC90 and BpSRK2A were incubated together for phosphorylation assays. There was no phosphorylated protein detected when only BpNAC90 was incubated; whereas BpNAC90 phosphorylation could be detected when BpSRK2A and BpNAC90 were incubated together, and phosphorylation level of BpNAC90 gradually increased with prolonged incubation time (Fig. 7E). These results indicated that BpNAC90 phosphorylation is mediated by BpSRK2A.

## Discussion

### BpNAC90 plays an important role in response to drought stress

We identified that *BpNAC90* was at the top level of a GRN responding to drought in a previous study, indicating that it might serve as an upstream regulator in mediating drought tolerance [32]. Here, our study showed that overexpression of *BpNAC90* conferred drought tolerance, whereas knocking out *BpNAC90* resulted in decreased drought tolerance (Fig. 1; Fig. S1A and B, see online supplementary material).

Increased proline levels can be induced in plants by various abiotic stresses. In response to abiotic stress, proline not only serves as a signal molecule, but also can enhance photosynthesis and non-enzymatic and enzymatic antioxidant activity, and regulate osmolyte concentrations and Na/K homeostasis [33, 34]. In this study, our results indicated that BpNAC90 could induce the proline biosynthesis gene *P5CR*, to increase proline biosynthesis. At the same time, BpNAC90 also could decrease the expression of *P5CDH2* to reduce the degradation of proline (Fig. 2F). These regulations lead to increased proline levels, which significantly improved drought tolerance (Fig. 2A and F).

ROS includes hydroxyl radicals (OH•), H_2_O_2_, singlet oxygen (^1^O2), and superoxide radicals (O2•-), which are important in plant acclimation to adverse environment. Excess ROS will cause heavy damage to membranes and cellular components [35]. However, low ROS levels serve as signal transduction components. ROS can integrate the other signaling events, such as plant hormones, to interplay each other, thereby orchestrating the plant's response to abiotic stresses and driving changes in transcriptomic, proteomic, and metabolic networks, leading to plant acclimation to abiotic stress [36, 37]. Therefore, controlling ROS to low level is important for adapting to abiotic stress. In addition, when compared with WT plants, POD and SOD activities were both enhanced significantly in OE plants, and significantly reduced in *nac* lines (Fig. 2B and C). At the same time, the H_2_O_2_ and ROS contents were significantly decreased in the OE lines, but significantly increased in the *nac* lines compared with those in the WT plants (Fig. 2D and E). These results suggested that BpNAC90 can mediate the ROS scavenging capability to improve drought tolerance.

### BpNAC90 binds to Eomes2, ABRE, and Tgif2 to regulate target gene expression

NAC TFs usually bind to NACRS motif with two discontinuous core sequences of ‘CATGTG’ and ‘CGTG’ [38]. In addition to binding to discontinuous sequences, NAC TFs can also bind to continuous core sequences such as ‘TTNCGT(G/A)’ [39], ‘TGACG’ [40], ‘C(A/G)CG(T/G)’ [41], ‘TAGTT’, and ‘GAATC’ [42], and SNBE motif ‘(T/A)NN(C/T)(T/C/G)TNNNNNNNA(A/C)GN(A/C/T)(A/T)’ [43]. Thus, NAC TFs might bind to various motifs to exert their functions. Herein, ChIP-Seq identified 16 conserved DNA motifs that potentially bound by BpNAC90 (Fig. 3A). Among them, three randomly selected motifs (Eomes2, ABRE, and Tgif2) were verified to bind to BpNAC90 by yeast one hybrid (Fig. 3B) and EMSA (Fig. 3C), indicating that although BpNAC90 might bind to various motifs, such as NACRS and SNEB, it also binds to motifs such as Eomes2, ABRE, and Tgif2 to regulate gene expression (Fig. S3A–C, see online supplementary material), which finally facilitates drought tolerance in birch. From the 16 identified DNA motifs potentially bound by BpNAC90 (Fig. 3A), only part of these motifs shares the conserved sequence of ‘CGT(GA)’, including the motifs HOXC11, ABRE, c-Myc, and HEB, and other DNA motifs do not share any conserved core sequences. Considering the sequences of other DNA motifs previously identified, it seems that the DNA motifs bound by NAC proteins should have more different types of conserved sequences, suggesting the complexity of NAC binding components. Therefore, comprehensive understanding of the DNA motifs bound by NAC proteins will facilitate understanding of the functions of NAC in depth.

### BpNAC90 confers drought tolerance by phosphorylation modification

Protein phosphorylation or dephosphorylation modification is the main kind of PTM, playing an essential role in protein functional adjustment. Previous studies showed that NAC proteins are phosphorylated in response to drought stress. When exposed to drought, ABA-produced H_2_O_2_ in maize will induce the expression of *ZmCCaMK* and *ZmNAC84*; subsequently, activated ZmCCaMK phosphorylates Ser-113 of ZmNAC84, which then activates its downstream gene to induce ABA-induced antioxidant defense [44]. Herein, we showed that phosphorylation of BpNAC90 is essential to mediate drought tolerance (Fig. 4A). Mutation of Ser 205 of BpNAC90 abolished its phosphorylation (Fig. 4B), which reduced the capability of mutated BpNAC90 (S^205^A) to confer drought tolerance (Fig. 4C). However, it still confers some drought tolerance, and plants overexpressing *S^205^As* still displayed higher drought tolerance than WT birch plants (Fig. 5A–D; Fig. S5, see online supplementary material). The results of qRT-PCR showed that phosphorylated BpNAC90 could improve the binding of BpNAC90 to certain motifs (Fig. 6A) and increased the activation of gene expression (Fig. 6B–E). Therefore, phosphorylation of BpNAC90 led to enhanced regulation of its target genes (Fig. 6B–E). However, when phosphorylation of BpNAC90 was abolished, it could still bind to the motifs, but this binding was not induced by drought stress, and the capability of S^205^A to activate gene expression was highly reduced but not abolished (Fig. 6). These results explained why S^205^A could confer reduced, but not abolished, drought tolerance compared with BpNAC90.

Our results showed that BpSRK2A can interact with BpNAC90 (Fig. 7A–C), and the expression levels of *BpSRK2A* and *BpNAC90* shared similar profiles, peaking within 3 h of drought stress (Fig. 7D). Furthermore, this interaction leads to the phosphorylation of BpNAC90 (Fig. 7E), which peaked at 1.5 h of drought stress (Fig. 4A). Therefore, we concluded that both BpSRK2A and BpNAC90 are induced by drought at the early stage (Fig. 7D), followed by their interaction (Fig. 7A–C), which catalyzes BpNAC90 phosphorylation (Fig. 7E), leading to binding of BpNAC90 to gene promoters to activate gene expression (Fig. 6; , see online supplementary materialFig. S3), ultimately facilitating drought stress tolerance.

### The working model of how BpNAC90 confers drought tolerance

Based on our study, a model of BpNAC90-mediated drought tolerance was proposed based on the above results (Fig. 7F). The expression of *BpNAC90* and *BpSRK2A* were induced by drought stress, and BpNAC90 then interacts with BpSRK2A to form a complex, leading to phosphorylation of BpNAC90. Phosphorylated BpNAC90 recognized different DNA motifs, including Eomes2, ABRE, and Tgif2, to control the expression of genes associated with drought response, such as *P5CR*, *P5CDH2*, *PODs*, and *SODs*. In addition, other drought tolerance genes, including *Gs1*, *Pslrpe*, *MK1*, *FAD*, and *ATP protein*, were also regulated by BpNAC90 (Tables S2and S3, see online supplementary material). The induction of the above genes improved the capability of ROS scavenging, and increased the proline content, leading to reduced ROS damage and increased osmotic adjustment ability, finally improving drought tolerance.

## Materials and methods

### Plant materials and growth conditions

The plantlets of WT birch were cultivated in woody plant medium (WPM) supplied with 2.5% (w·v^−1^) sucrose, 1 mg·L^−1^ 6-Benzylaminopurine (6-BA) and 0.6% agar in a bottle, or grew in soil and vermiculite mixture (v:v = 3:1) in pots in a greenhouse. All these plants grew under conditions of 65–70% relative humidity and a light/dark photoperiod of 14 h/10 h at a temperature of 25°C.

### Vector construction and gRNA sequence determination

The coding sequence (CDS) of *BpNAC90* fused with a Flag tag was cloned into the pROKII plasmid (35S:Flag-*BpNAC90*). CRISPR-GE (http://skl.scau.edu.cn/) was used to design different guide RNAs (gRNAs). The primers for the gRNA sequence were synthesized and cloned into pEgP237-2A-GFP (kanamycin resistance) for clustered CRISPR editing [45]. These constructs were transformed into *A. tumefaciens* (*Agrobacterium tumefaciens*) EHA105 for plant transformation. The gRNA with high Cas9 cleaving efficiency was determined using the TCEP method [46]. Briefly, transient transformation was performed following Zang *et al.* [47], DNA and RNA were respectively isolated from the transiently transformed plants for qPCR and qRT-PCR, and the primers were designed to amplify the region containing gRNA target sites. The specific sequences of *Bp5G13007*, *Bp1G24271*, *Bp12G11354*, *Bp9G16353*, and *Bp8G16690* (200 bp in length) were reversely and forwardly cloned into pFGC5941 to construct RNAi-silence vector, respectively. These constructs were transformed separately into *A. tumefaciens* EHA105. All the primers were shown as in Table S4 (see online supplementary material).

### Stable and transient genetic transformation

For stable transformation, the explants of birch were immersed in a solution containing 0.6 OD_600_ of the transformed Agrobacterium cells for 30 sec, excess Agrobacterium cells were removed using sterile filter paper, and the explants were cultured on a co-culture medium (WPM, pH 5.6 + 3% (w·v^−1^) sucrose +150 μM acetosyringone +1 mg·L^−1^ 6-BA) for 72 h. Thereafter, the explants were transferred to a differentiation medium (WPM, pH 5.8 + 3% (w·v^−1^) sucrose +350 mg·L^−1^ carbenicillin +1 mg·L^−1^ 6-BA +50 mg·L^−1^ kanamycin) for inducing callus and adventitious buds. When some plantlets were grown, they were moved to a medium for rooting (WPM, pH 5.8 + 0.2 mg·L^−1^ NAA +3% (w·v^−1^) sucrose +50 mg·L^−1^ kanamycin). The transient transformation was carried out following Zang *et al.* [47]. In brief, the plantlets were immersed in a solution for transformation (2 mM MES-KOH, pH 5.6 + 120 μM AS+ 10 mM CaCl_2_ + 2% sucrose+200 mg·L^−1^ DTT + 20 μM 5-Azacytidine +270 mM mannitol) containing 0.7 OD_600_ of *A. tumefaciens* cells, and shaken at 90 rpm at 25°C. After immersing for 60 min, 30 mL of fresh transformation solution was used to wash the plantlets quickly, and the plantlets were planted vertically on growth medium (1/2MS, pH 5.6 + 2% (w·v^−1^) sucrose+150 M acetosyringone +200 mg·L^−1^ DTT) for growth. After transient transformation for 36–48 h, the transiently transformed genes will express highly, and the plants can be used for study.

### Western blotting analysis

Total proteins or nuclear proteins were isolated from birch using a Plant Total Protein Extraction Kit or CelLytic™ PN Isolation/Extraction Kit (Sigma-Aldrich, St Louis, MO, USA) according to its user manual, and were separated with 12% (w·v^−1^) SDS-PAGE. After electrophoresis, proteins were transferred to PVDF membranes (Millipore, Billerica, MA, USA) by Trans-Blot Turbo Transfer System (Bio-Rad, Hercules, California, USA), using 5% skim milk to block, added anti-Flag antibodies, and incubated at 4°C for more than 6 h. Add secondary antibodies (horseradish peroxidase (HRP)-conjugated) and incubated for 1.5 h, and then added enhanced chemiluminescence (ECL) (Beyotime, Shanghai, China). The signals were screened using ECL prime (GE Healthcare, Chicago, IL, USA).

### Stress treatment and physiological analysis

To detect the growth phenotype, the plants grew in the soil without watering for 12 days (silica gel was first used to dry the soil), and the plants were watered with fresh water for rehydration. The growth phenotype was observed by photography, and the fresh weight, root weight, and root-top ratio were measured, respectively. For physiological analysis, the plantlets in pots were watered with a solution of 20% (w·v^−1^) PEG6000 directly on the roots thoroughly, and the plantlets well-watered with fresh water were served as control. The plantlets were harvested for physiological analysis after 7 days of treatment. Analysis of electrotype leakage and the MDA content were conducted according to the method of Zang *et al.* [48]. SOD and POD activities, and the ROS content, were measured, respectively, using the kits of copper-zinc superoxide dismutase assay, peroxidase assay and reactive oxygen species assay (Nanjing Jiancheng Bioengineering Institute, Nanjing, China). The H_2_O_2_ content was measured in accordance with the procedure described by Dal Santo *et al.* [49] and the proline content was determined according to the procedure described by Bates *et al.* [50]. Three independent biological replicates were carried out, and 10 plantlets of each line were used for each replicate. In total, 30 plantlets of each line were used.

### ChIP-seq and RNA-seq analysis

ChIP was performed using the birch plants overexpressing Flag-BpNAC90 (OE1) according to the procedures of Zhao *et al.* [51]. In brief, DNA and protein was cross-linked by 3% (v/v) formaldehyde, nuclei were isolated to extract DNA–protein crosslinks (DPCs). After sonication of DPCs into about 0.5Kb, DPCs were immunoprecipitated with an anti-Flag antibody, and DNA were released from DPCs by protease k digestion. For ChIP-seq assay, the reads were aligned to the genome of birch [52] using STAR software (Version 2.5.3a), peak calling was conducted using the MACS2 program (Version 2.1.1), and peaks distribution and annotation were analysed with the bedtools program (Version 2.25.0). MEME-ChIP was employed to determine the motifs within peaks using default parameters. The DEGs between the WT and OE1 plants using RNA-seq with three independent replicates. Both ChIP-Seq and RNA-seq were conducted by Seqhealth Technology Co., Ltd (Wuhan, China).

### Yeast one hybrid analysis

Three tandem copies of the sequences of the motifs, or their mutants, were cloned separately into pHIS2 (Clontech, Takara, China) as reporter vectors. The coding sequence (without termination codon) of *BpNAC90* was cloned into pGADT7-Rec2 (termed pGADT7-*BpNAC90*, effector). Yeast one hybrid screening was conducted following the user manual (BD Matchmaker™ Library Construction & Screening Kits User Manual). Yeast cells were plated on the medium TDO (SD/-His/−Leu/−Trp) containing 25 mM 3-Amino-1, 2, 4-triazole (3-AT) to select the positive clones. All primers used were listed in Table S5 (see online supplementary material).

### EMSA analysis

The maltose-binding protein (MBP) was fused with *BpNAC90* and cloned into pMAL-c5x prokaryotic expression vector (termed pMAL-BpNAC90-MBP) (NEB, USA). pMAL-BpNAC90-MBP was transformed into *Escherichia coli*, and 0.1 mM isopropyl b-D-1-thiogalactopyranoside (IPTG) was used to induce the expression of the fusion gene at 37°C. After induction for 2 h, BpNAC90-MBP was isolated using the pMAL protein fusion and purification system (NEB). The oligonucleotide DNAs containing the studied motif were synthesized, and biotin was conjugated at the 5′ terminus of these oligonucleotide (Sangon, China). The paired oligonucleotides were annealed forming double stranded DNA and were used as probes. Meanwhile, the oligonucleotides with the same sequence without a label were served as the competitors. The protein and probes were incubated for 2 h at room temperature and then subjected to PAGE electrophoresis. The binding of protein and DNA was determined with Chemiluminescent EMSA kit according to the protocol (Beyotime, Shanghai, China). All primers used are shown as Table S6 (see online supplementary material).

### ChIP analysis

ChIP was conducted following Zhao *et al.* [51]. Proteins and DNA were crosslinked with 3% (w·v^−1^) formaldehyde. After isolation and purification of nuclei, the chromatin was sonicated into 0.2- to 0.5- kb fragments. After sonication, the chromatins were immunoprecipitated with the anti-Flag antibody (ChIP+). The sonicated chromatin immunoprecipitated without any antibody was served as the negative control (ChIP-). The DNA and protein crosslink was decrosslinked with proteinase K, ChIP product was purified using a TIANgel Maxi Purification Kit (Qiagen, Hilden, Germany). The ChIP-PCR procedure was conducted as follows: 95°C 120 sec; 30 cycles of 94°C 25 sec, 58°C 25 sec, and 72°C 40 sec. All primers for ChIP-PCR are listed in Table S7 (see online supplementary material).

### Dual luciferase analysis

For analysing the regulation of BpNAC90 to its target genes, the promoters of target genes were respectively used to drive a *Luc* gene in pGreenII0800-LUC serving as reporter, and *BpNAC90* was driven by 35S promoter in pGreenII 62-SK (35S:*BpNAC90*) as effector. The effector and reporter constructs were respectively transformed into *A. tumefaciens* strain GV3101. Both effector and reporter together with the construct harboring 35S:Renilla LUC were cotransformed into birch plants following the method described by Zang *et al.* [47]. After 48 h of transformation, the transformed plantlets were transferred to a medium containing 20% PEG6000 and grew for 24 h. Dual LUC activities were determined with Firefly Luciferase Reporter Gene Assay Kit (Beyotime, Shanghai, China), and the activity of Renilla LUC was employed to normalize transformation efficiency, and LUC/REN ratio was calculated to determine the expression of genes regulated by BpNAC90. All primers used are shown in Table S4 (see online supplementary material).

### BpNAC90 phosphorylation analysis

The BpNAC90 transformed birch plants (OE) were treated with 20% PEG6000 for 0, 0.5, 1.5, 2, and 2.5 h, and the stressed plants were harvested for IP. Total proteins were isolated from birch by incubating with 3 ml of IP buffer (1 × PBS [phosphate-buffered saline] + 1% Triton X-100 + 1× phosphatase inhibitor+1× protease inhibitor) for 30 min at 25°C, and then were centrifuged at 15000 *g* for 10 min at 4°C twice. The supernatant was incubated with anti-Flag antibody (mAb) agarose (Sigma-Aldrich) for 3 h at 4°C to immunoprecipitate BpNAC90. After elution of the immunoprecipitated BpNAC90, the phosphorylated BpNAC90 was separated by SDS-PAGE and the phosphorylation was detected using western blotting using Phos-tag™ Biotin BTL-105 (WAKO Chemicals, USA). Phosphorylation modification of proteins can bind to Phos-tag Biotin that can be detected as ECL signals by reaction between horseradish POD and ECL substrate. Dephosphorylated BpNAC90 protein was prepared by treatment with calf intestinal alkaline phosphatase and served as a negative control.

### Identification of phosphorylation sites in BpNAC90

The potential phosphorylation site of BpNAC90 was predicted on the website NetPhos-3.1 (https://services.healthtech.dtu.dk/service.php?NetPhos-3.1). Four potential phosphorylation sites (Ser82, Ser141, Ser176, and Ser205) were predicted and were mutated by changing these codons from encoding serine to encoding alanine, separately. The mutated *BpNAC90s* were respectively cloned into pROKII by fusing with Flag-tag and were transiently and separately transformed into WT birch following the method of Zang *et al.* [47], and the transgenic plants transferred to a medium containing 20% PEG6000 for 1.5 h, and were harvested for study. Nuclear proteins were isolated from the transformed plants and the mutated BpNAC90 was isolated using anti-Flag antibody using IP. The isolated proteins were separated on 12% SDS-PAGE. The protein phosphorylation was determined using Phos-tag™ biotin HRP to determine the phosphorylated amino acid in BpNAC90. The *BpNAC90* gene with phosphorylation mutation was cloned into pROKII plant expression vector driven by 35S CaMV promoter and transformed into birch plants stably.

### The binding of BpNAC90 to motifs and target genes

The 46-bp minimal 35S promoter was fused with three tandem copies of the studied motif, cloned into pCAMBIA1301 vector to drive a *GUS* gene and served as reporters (Table S8, see online supplementary material). *BpNAC90* and its mutant (*S^205^A*) were respectively cloned into pROKII driving by 35S CaMV promoter as the effectors. Each effector (35S:*BpNAC90* or 35S:*S^205^A*) together with each reporter was transiently transformed into birch plants following Ji *et al.* [53]. At 2 days after transformation, these transient transgenic birches were subjected to drought treatment (20% PEG6000) for 1.5 h. The activation of BpNAC90 and S^205^A were determined by analysing GUS activity. The plants under normal conditions were used as the control. For normalization of the transformation efficiencies, 35S:*Luc* was also transformed together with the effector and reporter into birch.

### Quantitative RT-PCR and T7 endonuclease 1 (T7E1) analysis

Total RNA was isolated from birch using Plant RNA Kit (OMEGA) and digested with DNaseI (DNA free) to remove DNA. Total RNA (2 μg) was reversely transcribed into cDNA using oligo dT as primer with PrimeScript RT Reagent Kit (Takara, China) and was diluted to 100 μL as PCR templates. The reaction system of qRT-PCR or ChIP-qPCR was performed in 20 μL volume, contained reverse and forward primers (0.5 μM each), SYBR Green Real-time PCR Master Mix (Toyobo, Japan) (10 μL), and template cDNA (2 μL). The thermal profile was as follows: 94°C for 45 sec; then 45 cycles of 94°C for 15 sec, 58°C for 35 sec, and 72°C for 40 sec; in a qTower 2.2 system (Analytik Jena AG, Jena, Germany). The relative expression levels were calculated using the 2^–ΔΔCt^ method [54]. The DNA sequence of *TUB* (*Tubulin*) was used to normalize the quantity of cDNA in each reaction system. For ChIP-qPCR, the thermal profile was as follows: 94°C for 90 sec; 30 cycles of 94°C for 25 sec, 58°C for 35 sec, and 72°C for 44 sec. The primers used are listed in Tables S7 and S9 (see online supplementary material). T7E1 digestion was used to analyse the homozygous and heterozygous mutation induced by CRISPR system. The truncated chromatin containing the cleavage site of CRISPR was PCR amplified, heated at 96°C for 10 min to denature DNA, and heated at 68°C for 20 min to renature DNA. The renatured DNA was added with 1 μL of T7E1 (10 U·μL^−1^), incubated at 37°C for 20 min, and was visualized using agarose gel electrophoresis.

### Y2H analysis

The recombinant vectors were constructed using an In-Fusion HD Cloning Plus (Takara). The CDS of *BpNAC90* was cloned into pGBKT7 and transformed into Y2HGold yeast cells. The full-length cDNA library of birch was constructed using SMART™ technology (Switching Mechanism at the 5′ end of RNA Template; Takara). The SMART cDNA library (ds cDNA), linearized, and the pGADT7-Rec vectors were co-transformed into Y187 to form the library, respectively. The Y2HGold yeast cells harboring BpNAC90 and yeast Y187 cells harboring the cDNA library were co-transformed for hybridization, and co-cultured on the SD/−Trp-Leu medium and then selected on QDO medium (SD/−Leu-Trp-Ade-His) with 40 μg·mL^−1^ X-α-gal. All yeast cells on plates were cultured at 30°C for 3 days.

### Pull-down assays

The full-length CDS of *BpNAC90*-Strep tagII and *BpSRK2A*-Flag were cloned separately into vector pMAL-c5x (NEB, UK) and fused with MBP to obtain *BpNAC90*-MBP-Strep and *BpSRK2A*-MBP-Flag. The recombinant constructs were transformed into *E. coli* cells and then induced using 0.1 mM IPTG at 37°C for 2 h. The fused proteins of BpNAC90-MBP-Strep and BpSRK2A-MBP-Flag were isolated following the method described by pMAL protein fusion and purification system (NEB, UK), and then the purified proteins were incubated with protein-binding buffer at 4°C for 2 h. The products were incubated with Flag magnetic beads at 4°C for 0.5 h, and washed with binding buffer four to five times. The magnetic beads in aliquots were incubated with elution buffer, and then the eluted proteins were separated using SDS-PAGE and analysed with anti-Flag and anti-tagII antibodies using western blotting, respectively.

### CoIP assays

The full-length CDS of *BpNAC90* fused with Flag (Flag-*BpNAC90*) and *BpSRK2A* fused with Strep tagII (Strep-*BpSRK2A*) were cloned into vector pROKII vector to generate overexpression vectors (pROKII-Flag-*BpNAC90* and pROKII-Strep-*BpSRK2A*). The two recombinant constructs were together transiently transformed into birch plantlets [47]. After 48 h of transformation, CoIP was performed using Flag magnetic beads and Strep tagII magnetic beads, respectively, at 4°C for 3 h. Then the magnetic beads were washed using washing buffer four to five times and then the proteins were eluted. The eluted proteins were separated on SDS-PAGE gels and subjected to western blotting with anti-Flag antibody (for the product collected by Strep tagII magnetic beads) and anti-strep tagII antibody (for the product harvest by Flag magnetic beads), respectively.

### Determination of the phosphorylation of BpNAC90 and BpSRK2A

The constructs of *BpNAC90*-MBP-Strep and *BpSRK2A*-MBP-Flag were transformed into *E. coli* cells. After purification of BpNAC90-MBP and BpSRK2A-MBP protein, the same quantity of BpNAC90 and BpSRK2A proteins were incubated together for 0, 10, 30, 60, and 90 min with 10 mM ATP, respectively, and then the phosphorylation of BpNAC90 proteins was detected with western blotting using Phos-tag Biotin BTL-105. The protein with phosphorylation that can bind to Phos-tag Biotin can be observed using Chemiluminescent EMSA kit (Beyotime, Shanghai, China).

### Statistical analysis

Statistical analysis was conducted with SPSS version 26.0 (IBM SPSS Corp., Armonk, NY, USA) program. Student’s *t* test and multiple comparisons (least significant difference (LSD)) were used to data comparison. In all analyses, a *P* value of <0.05 indicated statistical significance. Three biological replicates were generated for the statistical analyses and error bars indicate the standard deviation (SD) from the three experiments.

### Accession numbers

All the GenBank numbers were shown as following: *P5CR* (GenBank number: PP234636); *P5CDH2* (GenBank number: PP234635); *POD1*-*POD7* (GenBank number: PP234638, PP234639, KP711297, KP711300, KP711301, KP7113002, KP711303); *SOD1*-*SOD3* (GenBank number: KP711292, KJ452333, PP234637); *Bp5G13007*, *Bp1G24271*, *Bp12G11354*, *Bp9G16353*, *Bp8G16690* (GenBank number: PP216168, PP216169, PP216170, PP216171, PP216172); *BpNAC90* (GenBank number: MW202042); *Tubulin* (GenBank number: MT163278); *BpSRK2A* (GenBank number: OR498778).

## Acknowledgements

This work was supported by the National Natural Science Foundation of China (No. 31971684 and No. 32171737), and the Heilongjiang Touyan Innovation Team Program (Tree Genetics and Breeding Innovation Team).

## Author contributions

Z.W. and Z.H. performed the experiments and analysed the data. Y.W. and X.S. designed the study, conceived the experiments and drafted the manuscript. C.G. and C.W. assisted with some of the experiments. Z.W., Z.H., and Y.W. wrote the manuscript. All authors read and approved the final version of manuscript.

## Data availability

All data are available within the article and supplementary information. ChIP-seq and RNA-seq data could be retrieved in the NCBI data libraries with the accession number PRJNA1034772 and PRJNA033815, respectively.

## Conflict of interest statement

The authors declare no conflicts of interest.

## Supplementary data

Supplementary data is available at *Horticulture Research* online.

## Supplementary Material

Web_Material_uhae061
