# Supplementary material for: Phosphorylation of birch BpNAC90 improves the activation of gene expression to confer drought tolerance
Source: Hortic Res. 2024 Feb 28;11(4):uhae061. doi: 10.1093/hr/uhae061 (PMC11040210; doi:10.1093/hr/uhae061)
Supplement: Web_Material_uhae061 [file web_material_uhae061.zip › Supplementary information.docx]

**
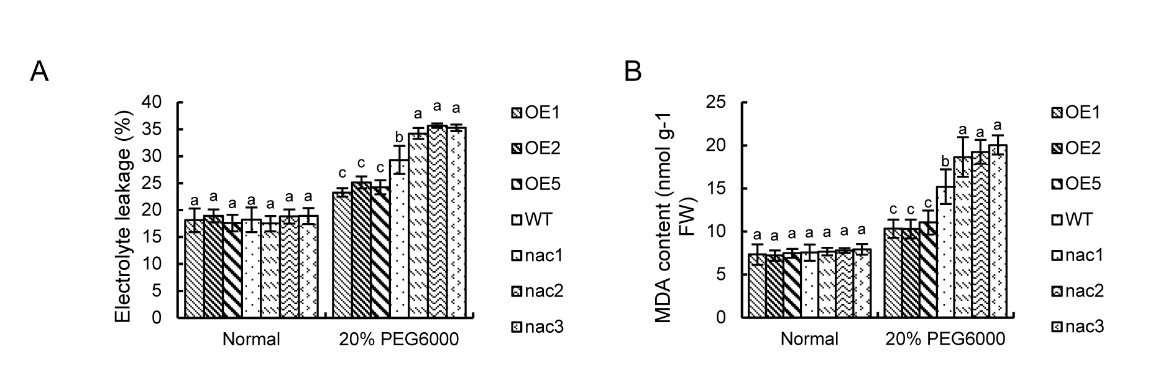
**

**Figure S1. Analysis of electrolyte leakage and MDA content.**

**A**, **B** Electrolyte leakage (**A**) and MDA content (**B**) analysis. The experiment was performed three times with similar results. Error bar indicates standard deviation (SD) from the three experiments. a - c indicate multiple comparison difference (LSD - t test, 0.05). OE1, 2 and 5: birch lines overexpressing *BpNAC90*; WT: wild-type birch; *nac1*-*3*: the plants with mutated *BpNAC90* induced by CRISPR/Cas9 birch lines.


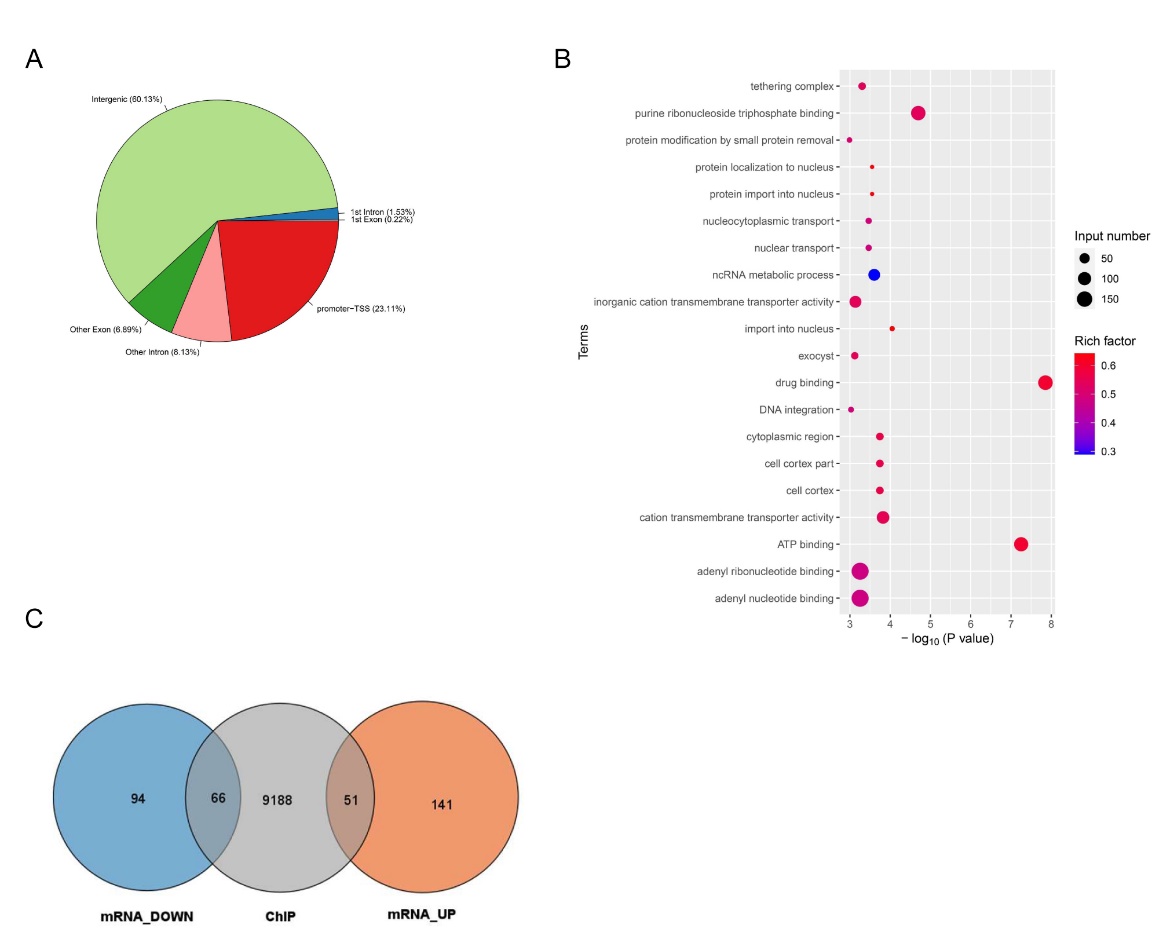


**Figure S2. Characterization of ChIP-seq.**

**A** The distribution of peaks in different functional genomic regions. **B** GO annotation of the genes’ peaks associated with peaks. **C** The Venn diagram for the genes distribution between ChIP-Seq and RNA‑seq.


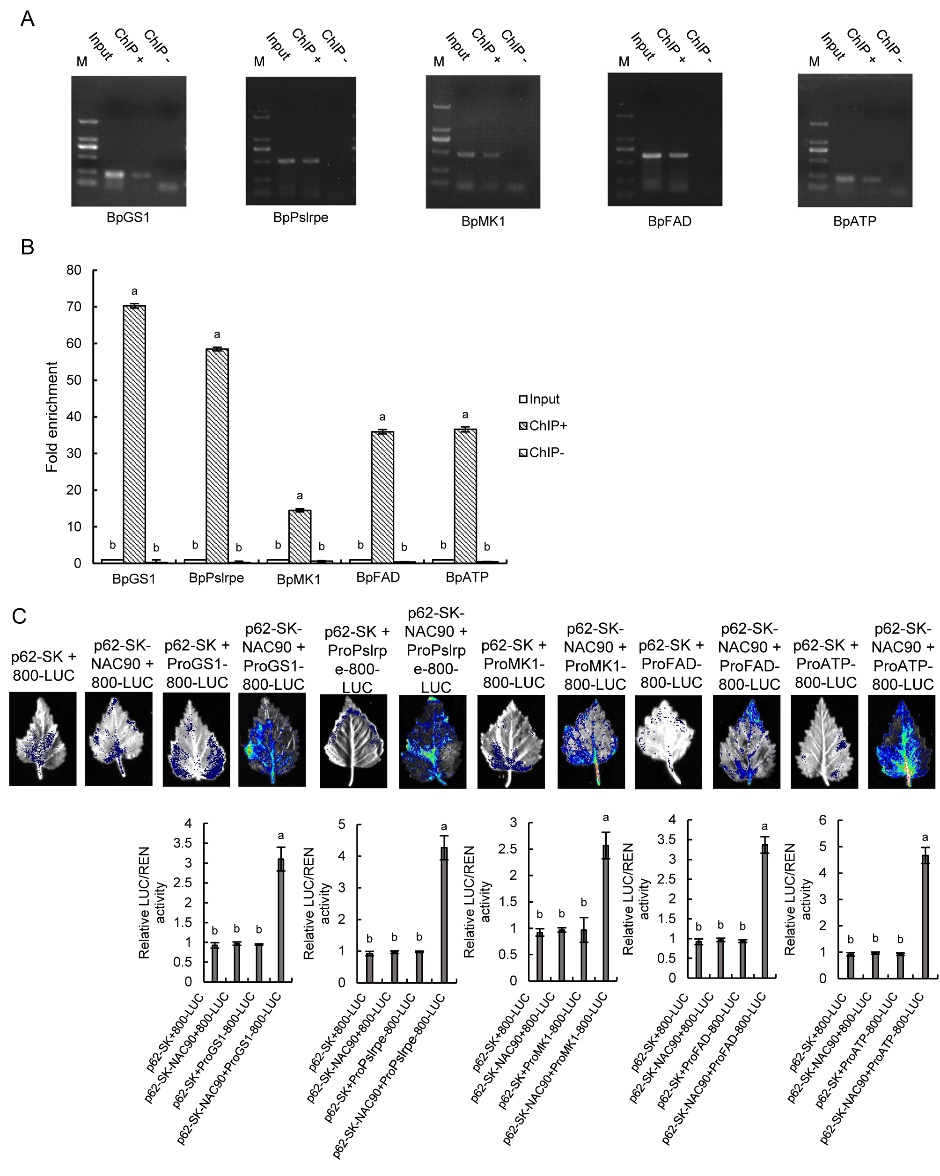


**Figure S3. Verification of ChIP-seq results.**

**A**, **B** ChIP-PCR (**A**) and ChIP-qPCR (**B**) were performed to determine the binding of BpNAC90 to the promoters of genes identified by ChIP-seq. Fold enrichment: the amount of aim DNA region relative to that of internal control DNA region, and fold enrichment in Input is set as 1 to calculate the relative fold enrichment in ChIP+ and ChIP-. **C** Dual-Luciferase Reporter analysis of activation expression of five genes bound by BpNAC90 on birch. p62-SK: empty pGreenII 62-SK vector; 800-LUC: empty pGreenII 0800-Luc vector; p62-SK-NAC90: BpNAC90 was cloned into pGreenII 62-SK under the control of 35S CaMV promoter; ProGS1/Pslrpe/MK1/FAD/ATP-800-LUC: the promoters of the five target genes was cloned into pGreenII 0800-Luc vector to drive the expression of LUC. 35S: Ren vector was transformed together to normalize the transient transformation efficiency. The experiment was performed three times with similar results. Error bar indicates standard deviation (SD) from the three experiments. a and b indicate multiple comparison difference (LSD - t test, 0.05).


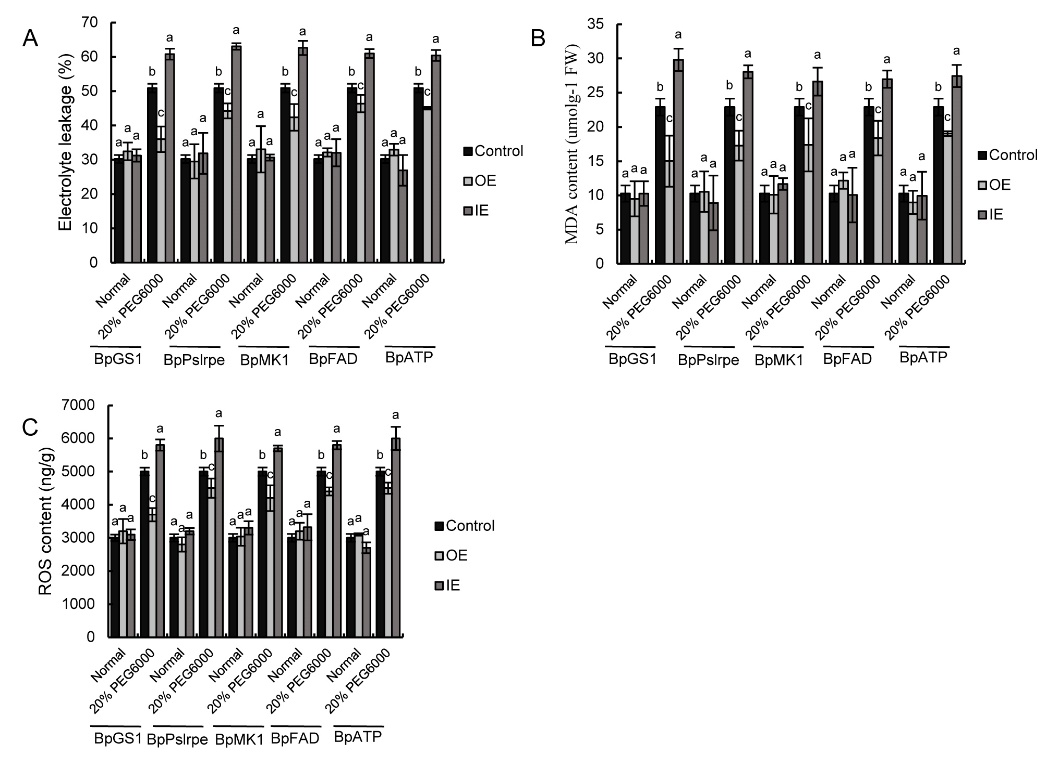


**Figure S4. Drought tolerance of the genes targeted by BpNAC90 identified by ChIP-Seq.**

**A** Electrolyte leakage rate analysis. **B** MDA content analysis. **C** ROS content analysis. Five genes targeted by BpNAC90 for identifying ChIP-Seq were selected for drought tolerance. Control: birch transformed with empty pROKII; OE: birch lines of 5 genes overexpression; IE: birch lines of RNAi-silenced expression. The experiment was performed three times with similar results. Error bar indicates standard deviation (SD) from the three experiments. a, b and c indicate multiple comparison difference (LSD - t test, 0.05).


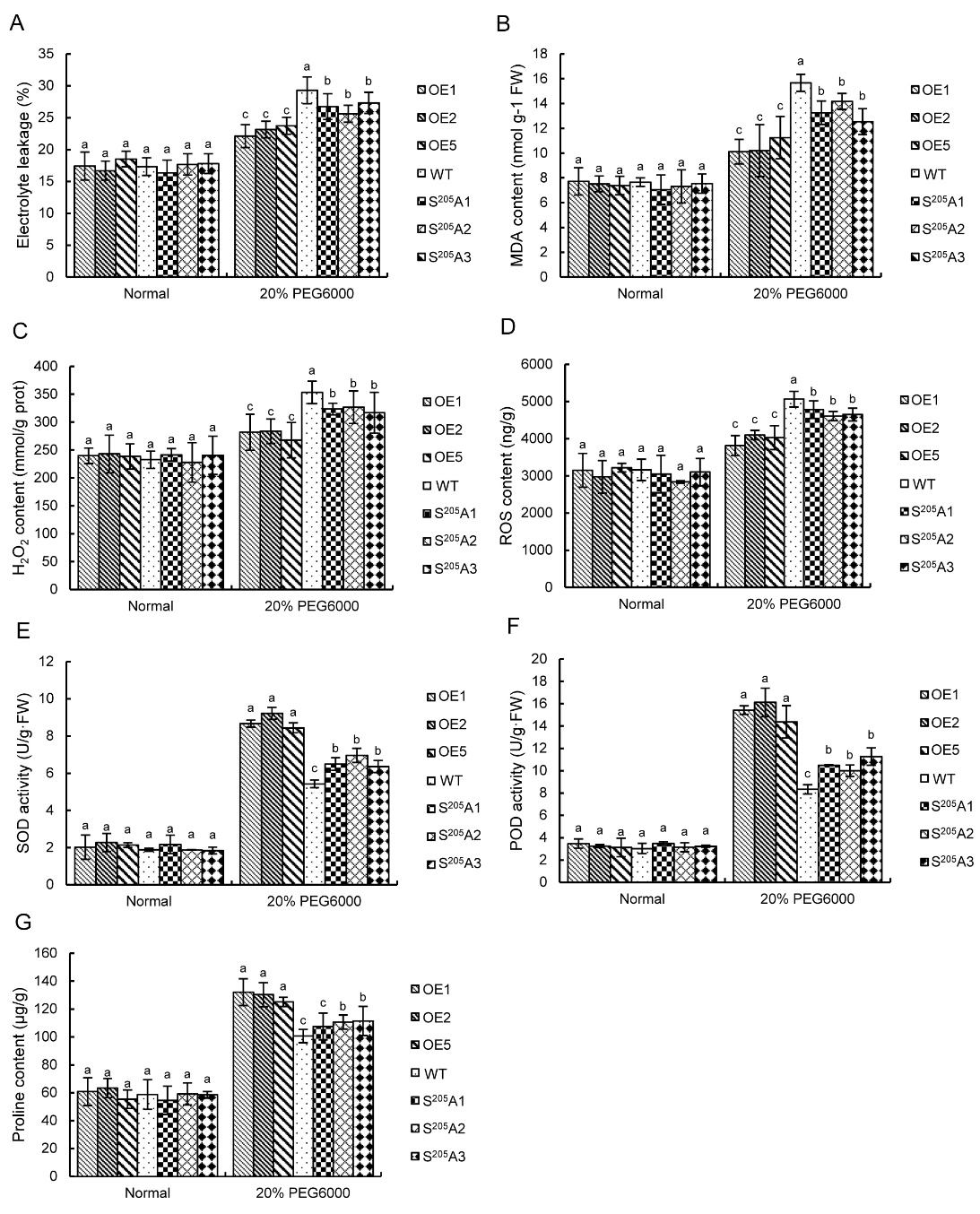


**Figure S5. Analysis of the drought tolerance of the *BpNAC90* mutated gene *S^205^A*.**

**A** Electrolyte leakage rate analysis. **B** MDA content analysis. **C** H_2_O_2_ content analysis. **D** ROS content analysis. **E** SOD activity assay. **F** POD activity assay. **G** Proline content assay. The *BpNAC90* mutated gene *S^205^A* loss of capability of phosphorylation were transformed into birch plants. OE1, 2, and 5: The birch lines overexpressing *BpNAC90*; WT: wild-type birch; *S^205^A1*-*3*: The birch lines (1-3) transformed with *S^205^A* for overexpression. The experiment was performed three times with similar results. Error bar indicates standard deviation (SD) from the three experiments. a, b and c indicate multiple comparison difference (LSD - t test, 0.05).

**Table S4. The primers used for constructing plant expression vectors.**

Primer sequences for vector construction

| Gene names | Primer sequences (5’-3’);  F: Forward; R: Reverse |
| --- | --- |
| pROKII-NAC90 | F: CTCTAGAGGATCCCCATGGAGGAATTCGCACCAG  R: TCGAGCTCGGTACCCTTATCTCCCAGGTAATGCTTC |
| CRISPR-Bpnac90 | F: GATTGTAAAGCGGACACTGAGCAA  R: AAACTTGCTCAGTGTCCGCTTTAC |
| pROKII-5G13007 | F: CTCTAGAGGATCCCCATGGCTCCTCATGAGCTCG  R: GGAAATTCGAGCTCGGTACCCTTAAGCCGCTGATGGCG |
| pROKII-1G24271 | F: CTCTAGAGGATCCCCATGGCTGCATTCCAGTTAT  R: GGAAATTCGAGCTCGGTACCCTTAGTTGCCATCTCCAC |
| pROKII-12G11354 | F: CTCTAGAGGATCCCCATGGAGAATCCGGCAATAC  R: GGAAATTCGAGCTCGGTACCCCTAATGAGCCCTTACAA |
| pROKII-9G16353 | F: CTCTAGAGGATCCCCATGGAAGCAGCAGTGCATG  R: GGAAATTCGAGCTCGGTACCCTTAGGAAATGCTAAGCT |
| pROKII-8G16690 | F: CTCTAGAGGATCCCCATGGAGAAAAGAAGAAGAT  R: GGAAATTCGAGCTCGGTACCCTCAAAAACTGTGCAGT |
| Cis-5G13007 | F: CATGCCATGGCCAGCTTCTGTAAATCCCG  R: TTGGCGCGCCTGGCACTCCAGGATCAAAC |
| Cis-1G24271 | F: CATGCCATGGTTCTTGCTCTGCTTGTTGC  R: TTGGCGCGCCGCCTGCTTCAGAGCTGGCAC |
| Cis-12G11354 | F: CATGCCATGGATGGAGAATCCGGCAATAC  R: TTGGCGCGCCCGCATTGTCCTTGAGCAAAG |
| Cis-9G16353 | F: CATGCCATGGGCAGCAGTGCATGAAG  R: TTGGCGCGCCTGATGTTGTCGGAGGGAG |
| Cis-8G16690 | F: CATGCCATGGGAAGAAGATATAGACACAC  R: TTGGCGCGCCCCAAACCGGTTACTGCTC |
| Anti-5G13007 | F: CTAGTCTAGACCAGCTTCTGTAAATCCCG  R: CGCGGATCCTGGCACTCCAGGATCAAAC |
| Anti-1G24271 | F: CTAGTCTAGATTCTTGCTCTGCTTGTTGC  R: CGCGGATCCGCCTGCTTCAGAGCTGGCAC |
| Anti-12G11354 | F: CTAGTCTAGAATGGAGAATCCGGCAATAC  R: CGCGGATCCCGCATTGTCCTTGAGCAAAG |
| Anti-9G16353 | F: CTAGTCTAGAGCAGCAGTGCATGAAG  R: CGCGGATCCTGATGTTGTCGGAGGGAG |
| Anti-8G16690 | F: CTAGTCTAGAGAAGAAGATATAGACACAC  R: CGCGGATCCCCAAACCGGTTACTGCTC |
| 62SK-NAC90 | F: GAATTCGATATCAAGCTTATGGAGGAATTCGCACCAG  R: GCGTACCGAATTGGTACCTTATCTCCCAGGTAATGC |
| p800-5G13007-LUC | F: ATCGAATTCCTGCAGCCCCACAACAACATTATGCAAG  R: AGAACTAGTGGATCCCCCGGAGGCTTCGATGTGGGCGG |
| p800-1G24271-LUC | F: ATCGAATTCCTGCAGCCCGGTCTAGAGTCCGATGTGC  R: AGAACTAGTGGATCCCCCGCAATTTGTAAGCTCACCGT |
| p800-12G11354-LUC | F: ATCGAATTCCTGCAGCCCGCAGATAGCTAGCTTGCACC  R: AGAACTAGTGGATCCCCCCGGGCTAGAGTGAACAGTA |
| p800-9G16353-LUC | F: ATCGAATTCCTGCAGCCCCGAAGAAGGACAAGTGTGG  R: AGAACTAGTGGATCCCCCGTCTGAAGGCAGCCCATACG |
| p800-8G16690-LUC | F: ATCGAATTCCTGCAGCCCGCTGGACAGTCTAAGAG  R: AGAACTAGTGGATCCCCCCGTCGCTATCACAAGGGGG |

**Table S5. The primers used for Y1H.**

Primer sequences for pHIS2 vectors

| Gene names | Primer sequences (5’-3’);  F: Forward; R: Reverse |
| --- | --- |
| EOMES2 | F: AATTCAACACCAACACCAACACCGAGCT  R: CGGTGTTGGTGTTGGTGTTG |
| EOMES2-1 | F: AATTCACCACCACCACCACCACCGAGCT  R: CGGTGGTGGTGGTGGTGGTG |
| EOMES2-2 | F: AATTCAAGACCAAGACCAAGACCGAGCT  R: CGGTCTTGGTCTTGGTCTTG |
| EOMES2-3 | F: AATTCACGACCACGACCACGACCGAGCT  R: CGGTCGTGGTCGTGGTCGTG |
| ABRE | F: AATTCCACGTGCACGTGCACGTGGAGCT  R: CCACGTGCACGTGCACGTGG |
| ABRE1 | F: AATTCCATGTGCATGTGCATGTGGAGCT  R: CCACATGCACATGCACATGG |
| ABRE2 | F: AATTCCACATGCACATGCACATGGAGCT  R: CCATGTGCATGTGCATGTGG |
| ABRE3 | F: AATTCCATATGCATATGCATATGGAGCT  R: CCATATGCATATGCATATGG |
| Tgif2 | F: AATTCTGTCATGTCATGTCAGAGCT  R: CTGACATGACATGACAG |
| Tgif2-1 | F: AATTCTGACATGACATGACAGAGCT  R: CTGTCATGTCATGTCAG |
| Tgif2-2 | F: AATTCTGCCATGCCATGCCAGAGCT  R: CTGGCATGGCATGGCAG |
| EOMES2M1 | F: AATTCCACACCCACACCCACACCGAGCT  R: CGGTGTGGGTGTGGGTGTGG |
| EOMES2M2 | F: AATTCACTCCCACTCCCACTCCCGAGCT  R: CGGGAGTGGGAGTGGGAGTG |
| EOMES2M3 | F: AATTCAACATCAACATCAACATCGAGCT  R: CGATGTTGATGTTGATGTTG |
| EOMES2M4 | F: AATTCAACACTAACACTAACACTGAGCT  R: CAGTGTTAGTGTTAGTGTTG |
| ABREM1 | F: AATTCTACGTGTACGTGTACGTGGAGCT  R: CCACGTACACGTACACGTAG |
| ABREM2 | F: AATTCCCCGTGCCCGTGCCCGTGGAGCT  R: CCACGGGCACGGGCACGGGGG |
| ABREM3 | F: AATTCCATGGGCATGGGCATGGGGAGCT  R: CCCCATGCCCATGCCCATGG |
| ABREM4 | F: AATTCCATGTTCATGTTCATGTTGAGCT  R: CAACATGAACATGAACATGG |
| Tgif2M1 | F: AATTCGGTCAGGTCAGGTCAGAGCT  R: CTGACCTGACCTGACCG |
| Tgif2M2 | F: AATTCTATCATATCATATCAGAGCT  R: CTGATATGATATGATAG |
| Tgif2M3 | F: AATTCTGTTATGTTATGTTAGAGCT  R: CTAACATAACATAACAG |

Primer sequences for pGADT7 vector

| Gene names | Primer sequences (5’-3’);  F: Forward; R: Reverse |
| --- | --- |
| pGADT7-NAC90 | F: GGCCATTATGGCCCGGGATGGAGGAATTCGCACCAG  R: GACATGTTTTTTCCCGGGTTATCTCCCAGGTAATGCTTC |

**Table S6. The primers used for constructing the prokaryotic expression vectors and amplifying DNA probes of EMSA.**

The primers used in construction of the prokaryotic expression vectors

| Gene names | Primer sequences (5’-3’);  F: Forward; R: Reverse |
| --- | --- |
| C5x-NAC90 | F: GGAATTCCATATGATGGAGGAATTCGCACCAGGT  R: CGCGGATCCTCTCCCAGGTAATGCTTC |
| C5x-SRK2A | F: GGAATTCCATATGATGGAGAAGTACGAGCTTG  R: GGAATTCTCACGAGGCATAGTTCCC |

Primer sequences for EMSA DNA probes

| Gene names | Primer sequences (5’-3’);  F: Forward; R: Reverse |
| --- | --- |
| EMSA-EOMES2 | F: AACACCAACACCAACACC (5’biotin)  R: GGTGTTGGTGTTGGTGTT (5’biotin) |
| EMSA-ABRE | F: CACGTGACACGTGACACGTG (5’biotin)  R: CACGTGTCACGTGTCACGTG (5’biotin) |
| EMSA-Tgif2 | F: TGTCATGTCATGTCA (5’biotin)  R: TGACATGACATGACA (5’biotin) |

**Table S7. The primers for analyses of ChIP-qPCR and ChIP-PCR.**

Primer sequences for ChIP-PCR

| Gene names | Primer sequences (5’-3’);  F: Forward; R: Reverse |
| --- | --- |
| ChIP-Bp5G13007 | F: CACAACAACATTATGCAAG  R: GTAAACTTGGGGTCTAAATC |
| ChIP-Bp1G24271 | F: GTGACCCAACACAACTCGC  R: GCAATTTGTAAGCTCACCGTG |
| ChIP-Bp12G11354 | F: GGACAACCCAACCTAGTA  R: CGGGCTAGAGTGAACAGTAGGC |
| ChIP-Bp9G16353 | F: CGAAGAAGGACAAGTGTGG  R: GTTTATCCCTTAAGAAGGTC |
| ChIP-Bp8G16690 | F: GCTGGACAGTCTAAGAG  R: GCCCTTTGGCTCAAGGATATTATC |
| ChIP-SOD1 | F: ATGCTATCTCTATCTTCTTTTC  R: CAATCTCTTTGTATTTATCAATAGC |
| ChIP-SOD2 | F: TCATAGAATGAGTAAGCTAT  R: GCGTAATTATATATGTTATCC |
| ChIP-POD1 | F: CTAATGAGGTGGTTCGGCC  R: CGAGAACCAAACAAGGCG |
| ChIP-POD7 | F: GGGTTGCAGACATTTAGTTGATC  R: GCGTCAATCCAGAAGGTTC |
| ChIP-P5CR | F: ATCACTTGGGATGGAAACCAAG  R: CCCCATTGGGGCAAAACGTGTG |
| ChIP-SOD1 (without motif) | F: GAGATTATGTTATTAAGTGAG  R: GAATACAAATATGAAATGGGACGTG |
| ChIP-SOD2 (without motif) | F: GAACGCAACTGGTGGATGTG  R: CTTGAATTCATATGTCAAT |
| ChIP-POD1 (without motif) | F: GCCGTTGCCCAGTCAAACAAG  R: ATATATAAGCATGAATGAAACATC |
| ChIP-POD7 (without motif) | F: CACTTCTCATCACTTCTTGC  R: ATTTCCCCATGTGAAGAA |
| ChIP-P5CR (without motif) | F: AGTGACAAAATAGATAGGGAT  R: AATAACTGTACATTCAGAGC |

Primer sequences for ChIP-qPCR

| Gene names | Primer sequences (5’-3’);  F: Forward; R: Reverse |
| --- | --- |
| Tubulin | F: TCAACCGCCTTGTCTCTCAGG  R: GGTTGTCACGTAAGCTCGGT |
| ChIP-q5G13007 | F: CACAACAACATTATGCAAG  R: GTAAACTTGGGGTCTAAATC |
| ChIP-q1G24271 | F: GTGACCCAACACAACTCGC  R: GCAATTTGTAAGCTCACCGTG |
| ChIP-q12G11354 | F: GGACAACCCAACCTAGTA  R: CGGGCTAGAGTGAACAGTAGGC |
| ChIP-q9G16353 | F: CGAAGAAGGACAAGTGTGG  R: GTTTATCCCTTAAGAAGGTC |
| ChIP-q8G16690 | F: GCTGGACAGTCTAAGAG  R: GCCCTTTGGCTCAAGGATATTATC |
| ChIP-qSOD1 | F: ATGCTATCTCTATCTTCTTTTC  R: CAATCTCTTTGTATTTATCAATAGC |
| ChIP-qSOD2 | F: TCATAGAATGAGTAAGCTAT  R: GCGTAATTATATATGTTATCC |
| ChIP-qPOD1 | F: CTAATGAGGTGGTTCGGCC  R: CGAGAACCAAACAAGGCG |
| ChIP-qPOD7 | F: GGGTTGCAGACATTTAGTTGATC  R: GCGTCAATCCAGAAGGTTC |
| ChIP-qP5CR | F: ATCACTTGGGATGGAAACCAAG  R: CCCCATTGGGGCAAAACGTGTG |

**Table S8. The primers for constructing pCAMBIA1301 vectors.**

Primer sequences for pCAMBIA1301 vector

| Gene names | Primer sequences (5’-3’);  F: Forward; R: Reverse |
| --- | --- |
| EOMES2-46 | F: GATCCCAACACCCAACACCCAACACCA  R: AGCTTGGTGTTGGGTGTTGGGTGTTGG |
| ABRE-46 | F: GATCCCACTGTCACTGTCACTGTA  R: AGCTTACAGTGACAGTGACAGTGG |
| Tgif2-46 | F: GATCCTGTCATGTCATGTCAA  R: AGCTTTGACATGACATGACAG |

**Table S9. The primers used for qRT-PCR.**

Primer sequences for qRT-PCR

| Gene names | Primer sequences (5’-3’);  F: Forward; R: Reverse |
| --- | --- |
| Tubulin | F: TCAACCGCCTTGTCTCTCAGG  R: GGTTGTCACGTAAGCTCGGT |
| RT-qNAC90 | F: AGAAATCCAACGGGTTATCCC  R: GAATACACGTAGCCAGGAGATCC |
| RT-q5G13007 | F: GAGAAAAGAAGAAGATAT  R: CCGGTTACTGCTCCTAATG |
| RT-q1G24271 | F: CATTCCAGTTATCTCTATC  R: CTCGAGACCCAGCATATGAG |
| RT-q12G11354 | F: GAGAATCCGGCAATACTG  R: CCGAAAATTGTCCCCGTCG |
| RT-q9G16353 | F: TGGAAGCAGCAGTGCATG  R: CATTCCCAGAAAGCCGTTG |
| RT-q8G16690 | F: TGGAGAAAAGAAGAAGATA  R: GGCACCAACCAAACCGGTTA |
| RT- qSOD1 | F: GCAGAGATCATGCAACTC  R: CGAGTGGTTTATATGACCTC |
| RT- qSOD2 | F: TGCTCCTAAAGCGGTGGTTC  R: GGGTTGAAATGAGGGCCAGT |
| RT- qSOD3 | F: ATGTAGGTCTGGTGGTGCTTC  R: AGAGAAGTGTGTGGATGGCTC |
| RT- qPOD1 | F: GGAGAGAAGAATGCAGCTC  R: GCTTGCAGTCCTAGCATCTCTTC |
| RT- qPOD2 | F: GAATGCAGCTCCCAATCGAAAC  R: GAATGCTGTTATTGGCAGCAGC |
| RT- qPOD3 | F: GCTGGGCCTAATGCTAATTC  R: GGAGGTCGGAATTAGCACC |
| RT- qPOD4 | F: TGAGGTGAATGGGAAGAATG  R: AGGTGATGTGGTTGGTGC |
| RT- qPOD5 | F: CAAGTCGGCAGTTGAGAAT  R: TGGAAAGGCCCACATTAC |
| RT- qPOD6 | F: ACATCTTAGCCGTTGTTGC  R: GGCAGTGAGACCTTTGTTT |
| RT- qPOD7 | F: TGTCAACAATTCTGGGCTAC  R: CCACCCTACAGTTCTTCCT |
| RT-qP5CR | F: CGAGCGAGACGTACAAGC  R: CCACATCACTGTCTTCAACC |
| RT-qP5CDH2 | F: TTCTATACCCTTTGCTACCG  R: GAATGCCTGTTTCATCTACC |
